# Single-cell transcriptomics reveals the cellular identity of a novel progenitor population crucial for murine neural tube closure

**DOI:** 10.1016/j.heliyon.2024.e37259

**Published:** 2024-08-30

**Authors:** Zihao Deng, Marina R. Carpinelli, Tariq Butt, Graham W. Magor, Peinan Zhao, Kevin R. Gillinder, Andrew C. Perkins, Stephen M. Jane

**Affiliations:** a Department of Medicine (Alfred Hospital), School of Translational Medicine, Monash University, 99 Commercial Rd, Melbourne, VIC, 3004, Australia; b Australian Centre for Blood Diseases, School of Translational Medicine, Monash University, 99 Commercial Rd, Melbourne, VIC, 3004, Australia; c QIMR Berghofer Medical Research Institute, 300 Herston Road, Brisbane, QLD, 4006, Australia; d Institute of Genetic Medicine and North-East England Stem Cell Institute, Centre for Life, Newcastle University, Central Parkway, Newcastle upon Tyne, NE1 3BZ, United Kingdom

**Keywords:** *Grhl3*, *Tfap2a*, *Tfap2c*, Neural tube closure, Neural tube defects, Single cell-RNA sequencing

## Abstract

Neural tube closure in vertebrates is achieved through a highly dynamic and coordinated series of morphogenic events involving neuroepithelium, surface ectoderm, and neural plate border. Failure of this process in the caudal region causes spina bifida. Grainyhead-like 3 (GRHL3) is an indispensable transcription factor for neural tube closure as constitutive inactivation of the *Grhl3* gene in mice leads to fully penetrant spina bifida. Here, through single-cell transcriptomics we show that at E8.5, the time-point preceding mouse neural tube closure, co-expression of *Grhl3*, *Tfap2a*, and *Tfap2c* defines a previously unrecognised progenitor population of surface ectoderm integral for neural tube closure. Deletion of *Grhl3* expression in this cell population using a *Tfap2a-Cre* transgene recapitulates the spina bifida observed in *Grhl3*-null animals. Moreover, conditional inactivation of *Tfap2c* expression in *Grhl3*-expressing neural plate border cells also induces spina bifida. These findings indicate that a specific neural plate border cellular cohort is required for the early-stage neurulation.

## Graphical abstract

**Figure:**
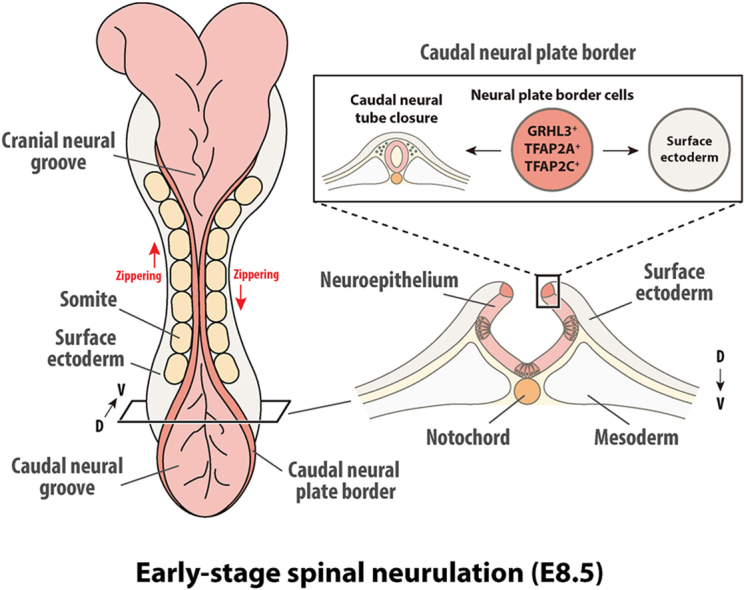

## 1 Introduction

Neural tube defects (NTDs) are a group of severe congenital malformations of the central nervous system that arise from neural tube dysmorphogenesis [1]. Open NTDs manifest as exposure of the spinal cord or brain due to failure in neural tube closure, a process in which the neural plate forms a closed tubular structure to provide a morphological scaffold for the development of the central nervous system [2]. Neural tube closure-related open NTDs are classified into three subtypes: anencephaly (failure of neural plate closure at the cranial region), spina bifida (failure of neural plate closure at the spinal region) and craniorachischisis (where the entire tube largely remains open along the cranial-caudal axis) [[3], [4], [5]]. Occurring between day 22 and 26 post-fertilisation in human and between embryonic day (E) 8.5 to E10.0 in mouse, neural tube closure is a critical developmental milestone in early embryogenesis [1,6]. Neural tube closure is achieved through a highly dynamic and coordinated series of morphogenic events in which a pair of apposed epithelial sheets progressively join together by ‘zippering’ along the cranial-caudal axis to create a hollow tubular structure [6]. This complex developmental process involves drastic cellular and molecular changes in neuroepithelium, the adjacent surface ectoderm and the interface of these two tissues, which is commonly referred to as the neural plate border.

Grainyhead-like 3 (GRHL3), a member of the family of GRHL transcription factors, is among one of the most crucial genes required for neural tube closure in both mouse and human [7,8]. Constitutive inactivation of its expression in murine embryogenesis leads to failures in dorsolateral hinge points formation and subsequently in caudal neural tube closure, resulting in fully penetrant thoraco-lumbo-sacral spina bifida. Additionally, *Grhl3*-null mice display occasional exencephaly, the embryonic precursor of anencephaly, soft-tissue syndactyly due to abnormal periderm morphogenesis, and a short longitudinal embryonic axis due to defects in the planar cell polarity pathway [7,[9], [10], [11]]. *Grhl3*-null mice die soon after birth of excessive transepidermal water loss due to defective skin barrier formation and show impaired keratinocyte differentiation and disrupted epidermal architecture [9,12]. Moreover, the curly tail (*ct*) mouse strain, an extensively utilised classical model of NTDs for more than five decades, carries a hypomorphic allele of *Grhl3* and displays incompletely penetrant caudal NTDs [7,13,14]. In humans, mutations in *GRHL3* have been identified as a major predisposing factor for developing spina bifida with several *de novo* and inherited variants in *GRHL3* reported in patients with NTDs [15,16].

Despite the identification of GRHL3 as an indispensable factor for neural tube closure, the molecular mechanisms by which GRHL3 induces this complex morphogenic process remains largely enigmatic. At E8.5, the developmental time-point preceding murine neural tube closure, *Grhl3* is highly and predominantly expressed along the caudal neural plate border, with expression expanding to the entire surface ectoderm by E10.5 [7,17,18]. In addition, *Grhl3* is expressed at a low level in the node-streak border (NSB) and caudo-lateral epiblast (CLE) regions, where neuromesodermal progenitors (NMPs) are found, and at low levels in the neuroepithelium at E8.5 [19]. Between E9.0-E9.5, *Grhl3* is expressed mosaically in neuroepithelium of the open posterior neuropore [13,19,20], and at E10.0-E10.5, expression is undetectable in neuroepithelium, but emerges within the hindgut endoderm [13,19]. De Castro et al. [19] have explored which of these *Grhl3* expression sites are critical for neural tube closure by specifically inactivating *Grhl3* expression in hindgut endoderm using the *Sox17-Cre*, in neuroepithelium and NMPs using the *Nkx1-2-Cre*, and in all these sites using both drivers. Only embryos with *Grhl3* expression inactivated in hindgut endoderm showed mild caudal NTDs with incomplete penetrance due to excessive caudal curvature [19]. Although this finding indicates that hindgut endoderm-specific *Grhl3* function is required for spinal neurulation, *Grhl3* is not expressed at this site until E10.0, suggesting that the early stage of neural tube morphogenesis must require *Grhl3*-expression elsewhere. Given that at E8.5, *Grhl3* expression is largely confined to the neural plate border territory [18], it is conceivable that loss of *Grhl3* expression at this site may underpin *Grhl3*-null caudal NTDs. This is in keeping with recent studies showing that loss of *Grhl3* expression is correlated with the loss of lamellipodia and other cellular structures in neural plate border cells that are critical for caudal neural tube closure [7,21,22].

The importance of the neural plate border cell population has also been defined in tissue explant studies in chicken. Selective removal of the neural plate border cells from explant chick neural plates *in vitro*, prevented neural plate folding and tube closure, despite successful midline longitudinal furrowing of the plate. However, if the neural plate border was left intact, even with the adjacent surface ectoderm removed, the neural plate folded and closed normally [[23], [24], [25]], indicating that the small cohort of neural plate border cells where *Grhl3* is expressed at E8.5 provides sufficient inductive and mechanical contributions for complete neural tube closure. In keeping with this, Jaffe and Niswander [21] showed that *Grhl3*-null mice did not display any patterning and integrity defect in surface ectoderm during spinal neurulation. In contrast, other studies have suggested that abnormal surface ectoderm development, coupled with altered mechanical properties underpin *Grhl3*-null NTDs [17,19,26], leaving significant uncertainty as to the critical population for *Grhl3*-induced neural tube closure.

To address this, we have now employed single-cell RNA sequencing (scRNA-seq) to investigate the transcriptomic profiles of specific cell populations in wild-type (WT) and *Grhl3*-null mouse embryos at E8.5 (the 6-7-somite stage). We found that *Grhl3* is co-expressed with transcription factor genes *Tfap2a* and *Tfap2c* in the neural plate border territory. RNA velocity analysis predicted that the neural plate border cell cohort marked by the co-expression of *Grhl3*, *Tfap2a*, and *Tfap2c* is a novel progenitor population of surface ectoderm. Deletion of a conditional allele of *Grhl3* using a mouse line expressing *Cre* recombinase expressed from *Tfap2a* regulatory elements recapitulated the spina bifida observed in constitutive *Grhl3*-null animals. Moreover, conditional inactivation of *Tfap2c* expression in *Grhl3*-expressing neural plate border cells also caused caudal neural tube defects, demonstrating a clear de-association between neurulation and surface ectoderm development. These findings suggest that at the initiation of neural tube closure, *Grhl3*-expressing neural plate border cells are neither committed surface ectoderm nor neuroepithelial/neural crest cells, but a previously unidentified progenitor population that plays a necessary and sufficient inductive role in the early-stage spinal neurulation.

## 2 Results

### 2.1 Single-cell transcriptomics profiling of E8.5 mouse embryo

**Fig. 1:**
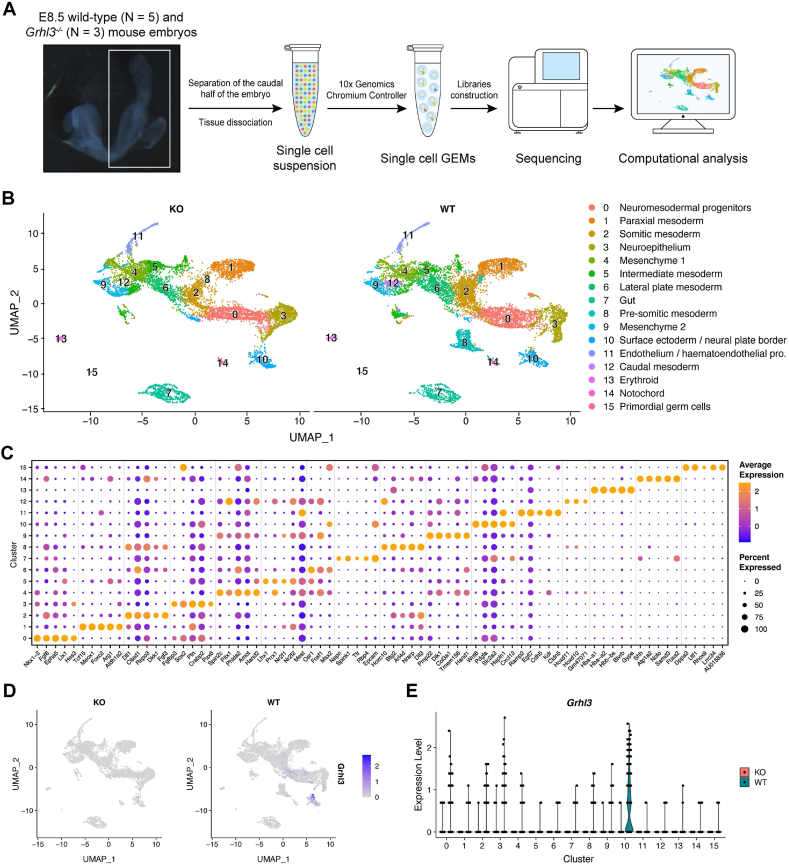
Single-cell RNA sequencing delineates diverse cell types in the caudal half of E8.5 mouse embryo. (A) Schematic diagram showing the workflow of scRNA-seq analysis on the caudal half of E8.5 wild-type (N = 5) and *Grhl3*^*−/−*^ (N = 3) embryos. (B) UMAP mapping of 20,196 cells from the scRNA-seq after quality control and filtering with unbiased clustering distinguished 16 cellular clusters. (C) Dot plot displaying expression profiles of top 5 differentially expressed genes from each cluster. The marker genes shared by multiple clusters are omitted from the plot. The size of a dot reflects the percentage of cells in a cluster that express the gene; the colour of a dot represents expression level of the gene. (D) UMAP showing the expression distribution and level of *Grhl3*. (E) Violin plot showing the expression distribution and level of *Grhl3*.

To examine the transcriptomic profiles of cell populations crucial for neural tube closure, we harvested E8.5 (the 6-7-somite stage) WT and *Grhl3*-null mouse embryos to generate single cell suspensions from the caudal half of these embryos for droplet-based scRNA-seq (Fig. 1A). At the sequencing depth of 400 million reads per library, we captured 24,239 cells with the average depth of 74,836 reads and 5537 genes detected per cell. After stringent quality control and filtering using Seurat, the final scRNA-seq dataset contained 20,196 cells. Unsupervised clustering identified 16 major cell types with distinct gene expression profiles that were projected onto Uniform Manifold Approximation and Projection (UMAP, Fig. 1B). Each cluster was evaluated and annotated based on a set of differentially expressed genes (DEGs) that are known to mark the specific cell population (Fig. 1C and Table S1) [27]. These 16 clusters encompass cell populations originating from all three germ layers and their major derivatives, including six mesoderm populations, two ectoderm clusters and the endoderm derived gut tube (Fig. 1B). This indicates normal germ layer specification in both WT and *Grhl3*-null embryos, in keeping with the unaffected embryonic patterning in the null animals [12]. Of note, the scRNA-seq also identified a cluster of NMPs (Cluster 0), a progenitor population that contributes to both ectodermal and mesodermal lineages post gastrulation [28]. Next, we sought to delineate the specific *Grhl3*-expressing cellular populations at this stage by interrogating its expression pattern in the WT dataset. In keeping with the prior expression analyses [18,19], *Grhl3* is predominantly expressed in the surface ectoderm and neural plate border population (Cluster 10), and at a much lower level in NMPs (Cluster 0) and neuroepithelium (Cluster 3) at E8.5 (Fig. 1D–E).

### 2.2 Single-cell transcriptomics reveals novel cell signatures of *Grhl3*-expressing cells

To further examine the cellular identity of the minute cohort of *Grhl3*-expressing neural plate border cells seen in Fig. 1D, we identified genes that are co-expressed with *Grhl3* in this cohort at E8.5. Firstly, we performed a gene co-expression correlation analysis on *Grhl3* in Cluster 10 (surface ectoderm and neural plate border) only. In total, 18 top associated co-expression genes were identified, of which 16 genes showed positive correlations while two genes showed negative correlations with *Grhl3* expression in Cluster 10 (Fig. S1A). Importantly, we noted that some of these genes are known to play important roles in neural tube closure, including *Cldn3* [29], *F2rl1* [30], *Gadd45a* [31], *Dlx5* [32], *Bmp5* [33], *Pax3* [34,35], and *Epha4* [36]. Interestingly, we also saw several genes in this list, including *Tfap2c* [37], *Sox2* [38], *Dlx5* [39], *Bmp5* [40], *Pax3* [41], and *Epha4* [42], that are required for the development of neural crest cells. These findings illustrate that the *Grhl3*-expressing cell population in Cluster 10 resides within a region that shows remarkable developmental complexity and multipotency.

Of the 18 top co-expression genes, one gene, *Tfap2c*, is a well-characterised gene that demarcates the neural plate border domain at this stage, in keeping with its indispensable role in neural crest induction [43]. In a mouse model, constitutive inactivation of *Tfap2c* expression led to early embryonic lethality between E7.5 and E8.5 due to defective extra-embryonic membrane and placenta development [44], while specific inactivation of *Tfap2c* expression in *Sox2*-expressing cells caused a low penetrant curly tail phenotype [45], suggesting that apart from neural crest development, *Tfap2c* may also play a role in neural tube closure. Of note, its duplicated paralog gene, *Tfap2a*, is also a gene that marks the neural plate border territory during neurulation [46]. The transcription factors encoded by these two paralogs are both required for early neural crest specification from neural plate border territory as shown in chicken, zebrafish, and *Xenopus* studies [37,[47], [48], [49]]. Interestingly, in contrast to *Tfap2c*-null animals, *Tfap2a*-null mouse embryos exhibited a profound exencephaly phenotype with curly tail [50,51]. Moreover, a recent study has shown that *Grhl3* and *Tfap2a* genetically interact to induce neural tube closure in mouse, as loss of one allele of both genes is sufficient to cause both cranial and caudal NTDs [51].

**Fig. 2:**
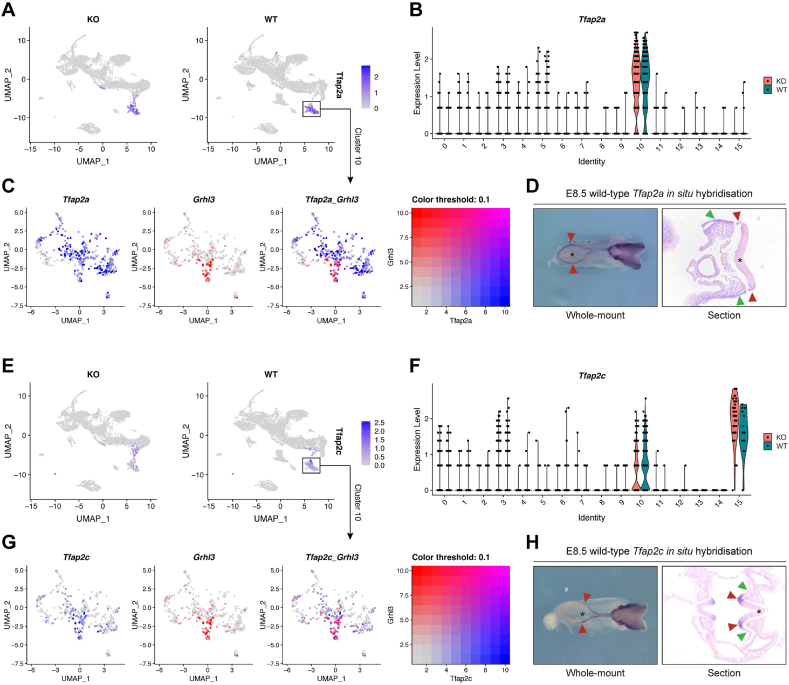
*Grhl3* is co-expressed with the classic hallmark genes of neural plate border, *Tfap2a* and *Tfap2c*, at E8.5. (A) UMAP showing the expression distribution and level of *Tfap2a*. (B) Violin plot showing the expression distribution and level of *Tfap2a*. (C) UMAP mappings of Cluster 10 only showing the co-expression of *Grhl3* with *Tfap2a*. (D) Whole-mount *in situ* hybridisation confirming the expression pattern of *Tfap2a* in E8.5 wild-type mouse embryo. Red triangles, neural plate border; green triangles, surface ectoderm; asterisk, neuroepithelium. (E) UMAP showing the expression distribution and level of *Tfap2c*. (F) Violin plot showing the expression distribution and level of *Tfap2c*. (G) UMAP mappings of Cluster 10 only showing the co-expression of *Grhl3* with *Tfap2c*. (H) Whole-mount *in situ* hybridisation confirming the expression pattern of *Tfap2c* in E8.5 wild-type mouse embryo. Red triangles, neural plate border; green triangles, surface ectoderm; asterisk, neuroepithelium.

We therefore examined the overall and Cluster 10-specific expression patterns of *Tfap2a* and *Tfap2c* in our scRNA-seq dataset. Our UMAP mapping clearly delineated the largely confined *Tfap2a* expression in Cluster 10 (Fig. 2A–B). Apart from a cohort of cells that co-express *Grhl3* and *Tfap2a*, however, there are additional cells that do not express *Grhl3* but also reside in this cluster and express *Tfap2a* (Fig. 2C). This is explained by our WISH showing *Tfap2a* expression was not only present in the neural plate border, but also widely distributed in surface ectoderm (Fig. 2D), whereas *Grhl3* is not at this timepoint [18]. Although *Tfap2c* is also expressed in primordial germ cells at this stage (Cluster 15, Fig. 2E–F), *Tfap2c* expression is largely co-localised with *Grhl3* expression in Cluster 10 (Fig. 2G). Most importantly, as opposed to *Tfap2a* which displays more widespread expression at E8.5, the *Tfap2c* expression domain is confined to the neural plate border territory, and no *Tfap2c* expression was detected in surface ectoderm (Fig. 2G–H). This expression pattern highly mirrors *Grhl3* expression in neural plate border at E8.5 [18]. Of note, the expression pattern and level of both *Tfap2a* and *Tfap2c* are normal in *Grhl3*-null embryos (Fig. 2B and F).

### 2.3 Co-expression of *Grhl3*, *Tfap2a*, and *Tfap2c* defines a previously unrecognised cell population

Given our finding of the co-expression of *Grhl3*, *Tfap2a*, and *Tfap2c*, and the fact that *Tfap2a* and *Tfap2c* are critical genes for neural crest specification from neural plate border, we next examined the expression patterns of several genes involved in neurulation and/or in neural crest development. Notably, our scRNA-seq showed that at E8.5, although *Grhl3*-expressing neural plate border cells express surface ectoderm progenitor markers *Krt8*, *Pdgfa*, *Epcam*, and *Wnt6*, they express neither committed surface ectoderm markers *Krt5* and *Trp63* nor neuroepithelium markers *Fgfbp2* and *Nkx6-1* (Fig. S1B). However intriguingly, these cells express the characteristic markers of neural crest progenitors and early neural crest genes *Sox2*, *Pax3*, *Pax7*, and *Tfap2b* (Figs. S1A–B) but not the neural crest cell specifiers *Sox9* and *Snai2* (Fig. S1B). This finding is surprising as the *Grhl3*-null animals exhibit normal neural crest development [7,9]. These findings suggest that *Grhl3* expression distinguishes a unique neural plate border cell population from committed surface ectoderm, neuroepithelium and neural crest identities, but defines a previously unrecognised population critical for neural tube closure.

To further examine this novel cell population marked by the co-expression of *Grhl3*, *Tfap2a* and *Tfap2c*, we applied RNA velocity analysis to our scRNA-seq dataset to dissect the gene-specific transcriptional and cellular dynamics within this cell cohort [52]. RNA velocity analysis measures the relative abundance of un-spliced nascent RNA and spliced mature RNA and uses this information to predict the differentiation dynamics of cells. In WT surface ectoderm and the neural plate border cluster, Velocyto identified a clear root of differentiation (Figs. S2A–B), which contributed to the surface ectoderm and neural plate border populations. In *Grhl3*-null embryos, it is apparent that this normal differentiation program has been significantly compromised, as evidenced by the ambiguous differentiation trajectories (Figs. S2A–B). Intriguingly, without *Grhl3* expression, the neural plate border cell cohort lost its neural plate border configuration and shifted its identity towards surface ectoderm (Figs. S2B–C). We next assessed the splicing patterns of *Grhl3*, *Tfap2a*, and *Tfap2c* transcripts in the surface ectoderm and neural plate border cluster by plotting the un-spliced residuals of each gene (Fig. S2D). In WT cells more un-spliced *Grhl3*, *Tfap2a*, and *Tfap2c* transcripts are located within or in the vicinity of the identified differentiation origin, whereas no similar significant pattern was identified in the constitutive null population (Fig. S2D). These RNA velocity estimates are also supported by the Monocle 3 analysis, which showed a differentiation trajectory from the *Grhl3*-expressing neural plate border cells to the surface ectoderm lineage (Fig. S3) [53]. Together, these predictions suggest that the neural plate border cell cohort marked by *Grhl3*, *Tfap2a*, and *Tfap2c* expression is a novel progenitor population of surface ectoderm.

### 2.4 Loss of GRHL3 in *Tfap2a*-expressing cells recapitulates all *Grhl3*-null defects

**Fig. 3:**
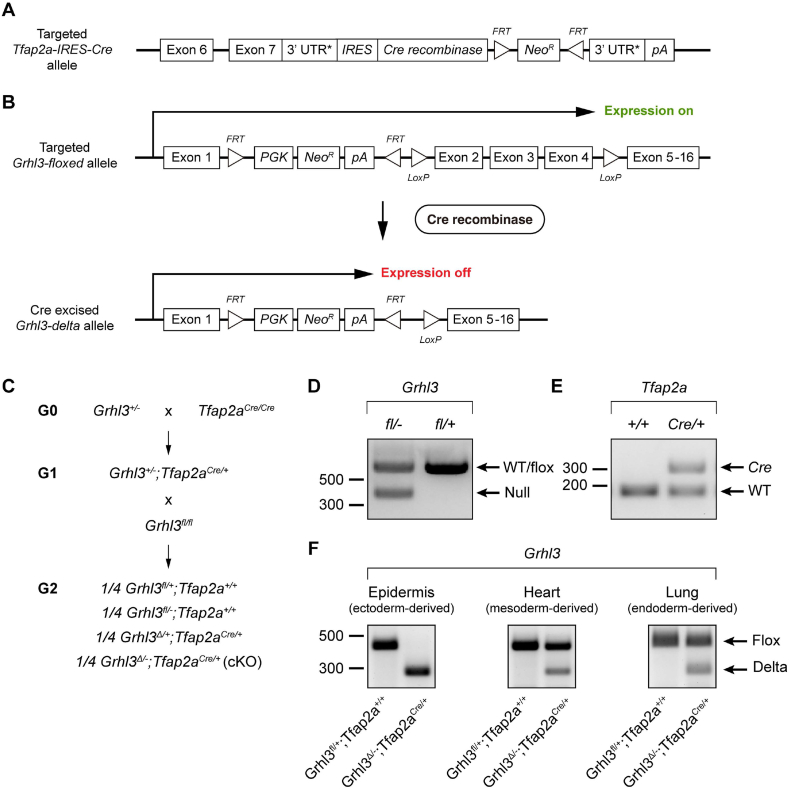
Generation of *Grhl3*^*flox*^*;Tfap2a*^*Cre*^ mouse model. (A) Schematic diagram showing the gene-targeting strategy for making the *Tfap2a-IRES-Cre* allele. The *IRES-Cre* cassette was inserted into the 3′UTR of *Tfap2a* locus. (B) When the *Tfap2a-Cre* is expressed, the *LoxP*-flanked *Grhl3* region, which contains exon 2 to exon 4, will be deleted. (C) Schematic diagram showing the breeding strategy for generating *Grhl3*^*flox*^*;Tfap2a*^*Cre*^ embryos. G0 *Grhl3* heterozygotes were crossed to *Tfap2a*^*Cre/Cre*^ mice to generate G1 double heterozygotes, which were then crossed to *Grhl3*^*fl/fl*^ mice to generate offspring with four different genotypes. (D) PCR genotyping of wild-type/floxed allele and null allele of *Grhl3* locus. (E) PCR genotyping of *Tfap2a-Cre* allele and wild-type *Tfap2a* allele. (F) PCR products of floxed allele and delta allele of *Grhl3* locus amplified from a representative organ or tissue derived from each of the three germ layers.

At E8.5, the time of the initiation of neurulation in mouse, *Grhl3* and *Tfap2a* display overlapping expression only in the neural plate border domain (Fig. 2C–D). We therefore used *Tafp2a* as a driver of the *Cre* recombinase gene expression to conditionally inactivate *Grhl3* in the neural plate border cell population. To achieve this, we crossed the *Grhl3*^*+/−*^ mice to a line in which the *Cre* recombinase gene has been integrated into the 3′UTR of the endogenous *Tfap2a* locus (*Tfap2a*^*Cre/Cre*^, Fig. 3A and C) [54]. This *Tfap2a-Cre* cassette also contains an *IRES* and is inserted upstream of the endogenous *Tfap2a* poly-adenylation signal, allowing the *Cre* recombinase gene to be expressed through *Tfap2a* regulatory elements with normal endogenous gene function unaffected (Fig. 3A) [54]. Previous lineage analysis has demonstrated that during neurulation, *Tfap2a-Cre* activity also occurs in ectoderm, especially in the pharyngeal arch ectoderm [54]. This cross generated a line heterozygous for both *Grhl3* and *Tfap2a-Cre* (*Grhl3*^*+/−*^*;Tfap2a*^*Cre/+*^), which was then crossed to a *Grhl3*^*fl/fl*^ line to generate *Cre*-heterozygotes carrying one *Grhl3* floxed allele with either a WT *Grhl3* allele or a *Grhl3*-null allele (Fig. 3B–E). With the presence of the Cre recombinase, the floxed *Grhl3* region which contains exon 2–4 was excised, generating a *Grhl3* delta allele in *Cre*-positive mice (*Grhl3*^*Δ/+*^*;Tfap2a*^*Cre/+*^ and *Grhl3*^*Δ/-*^*;Tfap2a*^*Cre/+*^, Fig. 3B–E). Analysis of genomic DNA from tissues derived from each germ layer indicated that a complete deletion of exons 2–4 of *Grhl3* was only observed in surface ectoderm (Fig. 3F).

**Fig. 4:**
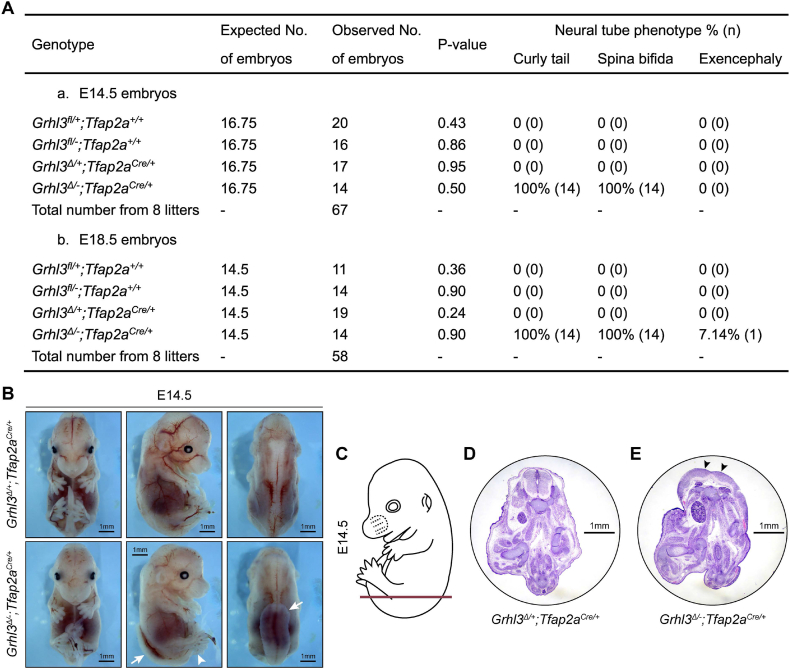
Specific inactivation of *Grhl3* expression in *Tfap2a*-expressing cells is sufficient to induce spinal neural tube defects. (A) Expected and observed numbers of embryos carrying corresponding genotypes from the cross of *Grhl3*^*+/−*^*;Tfap2a*^*Cre/+*^ and *Grhl3*^*fl/fl*^ mice at E14.5 and E18.5, and the penetrance of neural tube defects in these embryos. The expected numbers of embryos were calculated based on the expected frequency of each genotype using the total number of collected embryos at each timepoint. A one sample χ2 test was used to compare the expected and observed number of embryos. (B) The gross appearances of E14.5 *Grhl3*^*flox*^*;Tfap2a*^*Cre*^ embryos. The *Grhl3*^*Δ/-*^*;Tfap2a*^*Cre/+*^ cKO embryos showed curly tail (white arrowhead) and thoraco-lumbo-sacral spina bifida (white arrows). (C) Schematic illustration showing the lower spinal position of transverse sectioning of E14.5 embryos in (D–E). (D) Transverse sectioning of *Grhl3*^*Δ/+*^*;Tfap2a*^*Cre/+*^ control embryos at the lower spinal region showing a closed neural tube. (E) Transverse sectioning of *Grhl3*^*Δ/-*^*;Tfap2a*^*Cre/+*^ cKO embryos at the lower spinal region showing a convex neural plate (black arrowheads).

In E14.5 and E18.5, embryos with all possible genotypes (*Grhl3*^*fl/+*^*;Tfap2a*^*+/+*^, *Grhl3*^*fl/-*^*;Tfap2a*^*+/+*^, *Grhl3*^*Δ/+*^*;Tfap2a*^*Cre/+*^, and *Grhl3*^*Δ/-*^*;Tfap2a*^*Cre/+*^) were present in a normal Mendelian ratio, indicating an absence of early embryonic lethality (Fig. 4A). To assess the outcome of neural tube morphogenesis in *Grhl3*^*Δ/-*^*;Tfap2a*^*Cre/+*^ conditional knockout (cKO) animals, we firstly harvested embryos at E14.5 to examine their gross phenotype. As expected, *Grhl3*^*Δ/-*^*;Tfap2a*^*Cre/+*^ cKO embryos showed a complete penetrance of curly tail and thoraco-lumbo-sacral spina bifida, mirroring the NTDs observed in *Grhl3*-null embryos [7,9]. In contrast, *Grhl3*^*Δ/+*^*;Tfap2a*^*Cre/+*^ control embryos had normal neural tube closure (Fig. 4B). Transverse sectioning of *Grhl3*^*Δ/-*^*;Tfap2a*^*Cre/+*^ cKO embryos at the low thoracic and lumbosacral region showed a convex caudal neural plate (Fig. 4C–E), which also phenocopied the malformed neural plate observed in constitutive *Grhl3*-null embryos [7]. *Grhl3*^*Δ/-*^*;Tfap2a*^*Cre/+*^ cKO animals also showed a low frequency of exencephaly, again mirroring the constitutive knockout (Fig. 4A) [7]. Together with the genomic DNA analysis showing a complete deletion of the floxed *Grhl3* region only in surface ectoderm, these observations suggest that the expression of *Grhl3* at other sites (and in particular the hindgut endoderm) was not critical for early-stage spinal neurulation (Fig. 3F). To further characterise the spina bifida lesion observed in the *Grhl3*^*Δ/-*^*;Tfap2a*^*Cre/+*^ cKO animals, we harvested *Grhl3*^*Δ/-*^*;Tfap2a*^*Cre/+*^ cKO embryos at E18.5 to generate full-body skeletal preparations. The *Grhl3*^*Δ/-*^*;Tfap2a*^*Cre/+*^ cKO embryos mimicked constitutive *Grhl3*-null animals, showing striking abnormalities in the vertebral column, with the first splayed vertebral pedicle (SVP) observed between the eighth to the tenth thoracic vertebrae (T8 to T10) coupled with severe kyphosis (Figure S4A-B and E). Together, these findings indicate that inactivation of *Grhl3* expression in *Tfap2a*-expressing cells is sufficient to induce spinal NTDs of equivalent severity to those seen in embryos with constitutive knock-out of *Grhl3*.

**Fig. 5:**
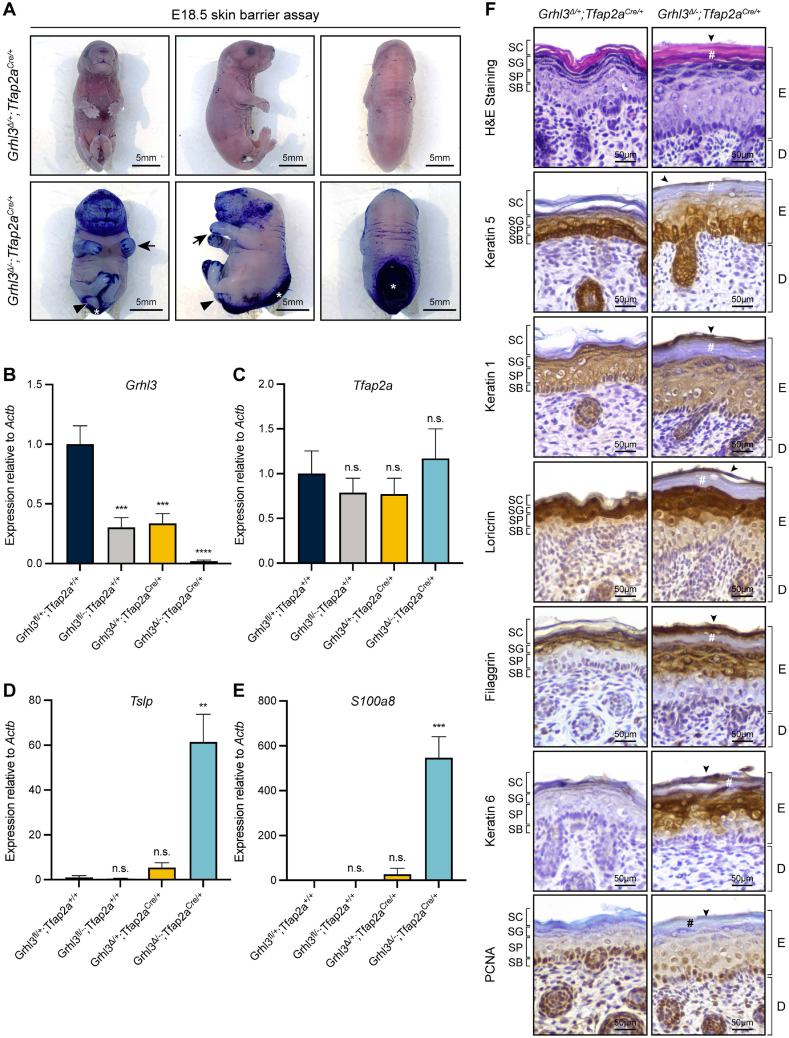
Specific inactivation of *Grhl3* expression in *Tfap2a*-expressing cells also leads to other *Grhl3*-null defects. (A) Skin barrier assay on E18.5 *Grhl3*^*flox*^*;Tfap2a*^*Cre*^ embryos. The *Grhl3*^*Δ/+*^*;Tfap2a*^*Cre/+*^ control embryos (N = 4) showed a fully formed epidermal barrier while the *Grhl3*^*Δ/-*^*;Tfap2a*^*Cre/+*^ cKO embryos (N = 6) exhibited a severe skin barrier defect as evident by the penetration of toluidine blue dye into skin. Black triangles, curly tail; black arrows, limb abnormalities; white asterisks, spina bifida. (B–E) Q-RT-PCR analyses on E18.5 epidermis showing the expression levels of *Grhl3*, *Tfap2a*, *Tslp* and *S100a8*. Bar graphs presented as mean ± standard error of mean. A one-way ANOVA test following by a Dunnett's multiple comparison test between *Grhl3*^*fl/+*^*;Tfap2a*^*+/+*^ embryos and other genotypes were used for data analysis. ** = P-value <0.01, *** = P-value <0.001, **** = P-value <0.0001. n.s., not significant. (F) Hematoxylin and eosin staining and immunohistology analysis of E18.5 *Grhl3*^*Δ/+*^*;Tfap2a*^*Cre/+*^ control and *Grhl3*^*Δ/-*^*;Tfap2a*^*Cre/+*^ cKO skin. N = 4. Black arrowheads, the extra tissue structure on the outermost layer of the epidermis; number signs, compacted stratum corneum layer. SC, stratum corneum; SG, stratum granulosum; SP, stratum spinosum; SB, stratum basale; E, epidermis; D, dermis.

Given that *Tfap2a* expression at E8.5 is not restricted to the territory of neural plate border, but is also highly expressed throughout the surface ectoderm (Fig. 2C–D), we examined whether the *Grhl3*^*flox*^*;Tfap2a*^*Cre*^ embryos exhibited defective epidermal development similar to the *Grhl3*-null animals. *Grhl3*^*Δ/-*^*;Tfap2a*^*Cre/+*^ embryos harvested at E18.5 demonstrated severely compromised skin barrier function, as assessed by external toluidine blue dye penetration [55], mimicking the profound skin barrier defect as seen in *Grhl3*-null animals (Fig. 5A) [7,9]. Apart from the impaired skin barrier and NTDs, *Grhl3*^*Δ/-*^*;Tfap2a*^*Cre/+*^ cKO embryos further phenocopied the *Grhl3*-null animals, showing a round body shape with a short craniocaudal axis, soft-tissue syndactyly and swollen limbs (Fig. 5A and S5A) [9,11,12,20]. Given these striking phenotypic similarities observed in the cKO animals, we isolated epidermis samples from E18.5 embryonic skin to evaluate mRNA expression levels of multiples epidermal genes. Consistent with the disrupted barrier formation, the expression level of *Grhl3* was reduced to a minimum (Fig. 5B) in *Grhl3*^*Δ/-*^*;Tfap2a*^*Cre/+*^ cKO epidermis, while the mRNA abundances of two markers for impaired epidermal integrity, *Tslp* and *S100a8*, were remarkably increased compared with the *Grhl3*^*fl/+*^*;Tfap2a*^*+/+*^ animals (Fig. 5D–E). The expression of *Tfap2a* was unaffected in *Grhl3*^*Δ/-*^*;Tfap2a*^*Cre/+*^ cKO embryos (Fig. 5C).

We next examined the architecture of epidermis from *Grhl3*^*Δ/+*^*;Tfap2a*^*Cre/+*^ control and *Grhl3*^*Δ/-*^*;Tfap2a*^*Cre/+*^ cKO animals at E18.5 using hematoxylin and eosin (H&E) staining and immunohistochemistry (IHC) analyses with various cell differentiation and proliferation markers. Strikingly, the *Grhl3*^*Δ/-*^*;Tfap2a*^*Cre/+*^ cKO epidermis was markedly thickened compared with that of *Grhl3*^*Δ/+*^*;Tfap2a*^*Cre/+*^ control embryos, mimicking the *Grhl3*-null epidermis (Fig. 5F) [9,12,56]. Similar to the abnormal epidermis observed in *Grhl3*-null mice, this thickening was coupled with a compacted stratum corneum (SC) layer and largely expanded stratum granulosum (SG) and stratum spinosum (SP) layers. The *Grhl3*^*Δ/-*^*;Tfap2a*^*Cre/+*^ cKO epidermis also showed an extra tissue structure attached to the SC layer on the surface of the skin, due to defective periderm desquamation caused by loss of GRHL3 [11]. Consistent with the abnormal epidermal architecture, IHC analysis showed that the expression domain of keratin (K) 5, an epidermal basal marker, was significantly expanded in the *Grhl3*^*Δ/-*^*;Tfap2a*^*Cre/+*^ cKO skin compared with the control (Fig. 5F). Similar abnormalities were observed with the suprabasal marker K1 and the terminal differentiation markers loricrin and filaggrin in the *Grhl3*^*Δ/-*^*;Tfap2a*^*Cre/+*^ cKO epidermis (Fig. 5F). Moreover, the epidermal stress and repair marker, K6, was highly upregulated in the *Grhl3*^*Δ/-*^*;Tfap2a*^*Cre/+*^ cKO epidermis while virtually absent in the *Grhl3*^*Δ/+*^*;Tfap2a*^*Cre/+*^ control skin (Fig. 5F). Examination of the proliferation marker, PCNA, showed a significantly increased positivity with expansion of PCNA-positive cells into the suprabasal layers of the *Grhl3*^*Δ/-*^*;Tfap2a*^*Cre/+*^ cKO epidermis, indicating basal keratinocyte hyperproliferation (Fig. 5F). These epidermal abnormalities phenocopied the constitutive *Grhl3*-null skin [9,12,56], indicating that inactivation of *Grhl3* in *Tfap2a*-exrpessing cells is also sufficient to perturb epidermal development.

### 2.5 Loss of TFAP2C in *Grhl3*-expressing cells causes neural tube defects

As *Tfap2a* expression overlaps with *Grhl3* expression in the surface ectoderm and epidermis during skin development [18,57], it is not surprising that loss of *Grhl3* expression in *Tfap2a*-positive cells recapitulated the constitutive *Grhl3*-null skin barrier defect. In view of this, we thought to use a more locally restricted driver to inactivate *Grhl3* specifically in neural plate border cells. As opposed to *Tfap2a*, which is far more prevalent at E8.5, *Tfap2c* expression is confined to the E8.5 neural plate border but not the surface ectoderm, which mirrors *Grhl3* expression pattern identically (Fig. 2G–H). Unfortunately, during mouse embryo development, the expression of *Tfap2c* also occurs early in developing oocytes, with a rapid decline immediately post fertilisation to be replaced by zygotic transcripts at the morula stage [58]. This suggested that using *Tfap2c* as a driver for *Cre* recombinase gene expression, would likely generate a constitutive *Grhl3* knockout, and we confirmed this by generating a *Tfap2c-Cre* mouse intercrossed with our *Grhl3*^*flox*^ line (Figure S4B and S6-S8).

**Fig. 6:**
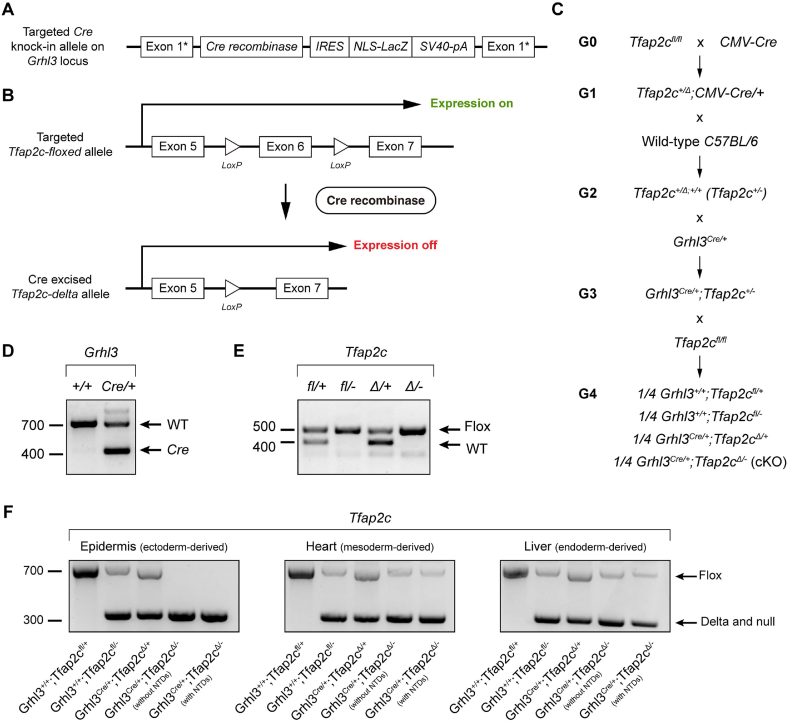
Generation of *Grhl3*^*Cre*^*;Tfap2c*^*flox*^ mouse model. (A) Schematic diagram showing the gene-targeting strategy for making the *Grhl3-Cre* allele. The *Cre* recombinase gene was inserted into the exon 1 of *Grhl3* locus. (B) With the expression of *Grhl3-Cre*, the floxed *Tfap2c* exon 6 will be deleted. (C) Schematic diagram showing the breeding strategy for generating *Grhl3*^*Cre*^*;Tfap2c*^*flox*^ embryos. G0 homozygous *Tfap2c*^*flox*^ mice were crossed to the *CMV-Cre* strain to generate the G1 *Tfap2c*^*Δ/+*^*;CMBV-Cre/+* line. This line was then then crossed to wild-type C57BL/7 mice to generate *Tfap2c*^*+/−*^ offspring. Further cross of the *Tfap2c*^*+/−*^ mice to the *Grhl3*^*Cre/+*^ line generated double heterozygous mice, which were timed mated with *Tfap2c*^*fl/fl*^ mice to produce embryos with four different genotypes. (D) PCR genotyping of the wild-type and *Cre* allele of *Grhl3* locus. (E) PCR genotyping of the wild-type and floxed allele of *Tfap2c* locus. (F) PCR products of floxed allele and delta allele of *Tfap2c* locus amplified from a representative organ or tissue derived from each of the three germ layers.

**Fig. 7:**
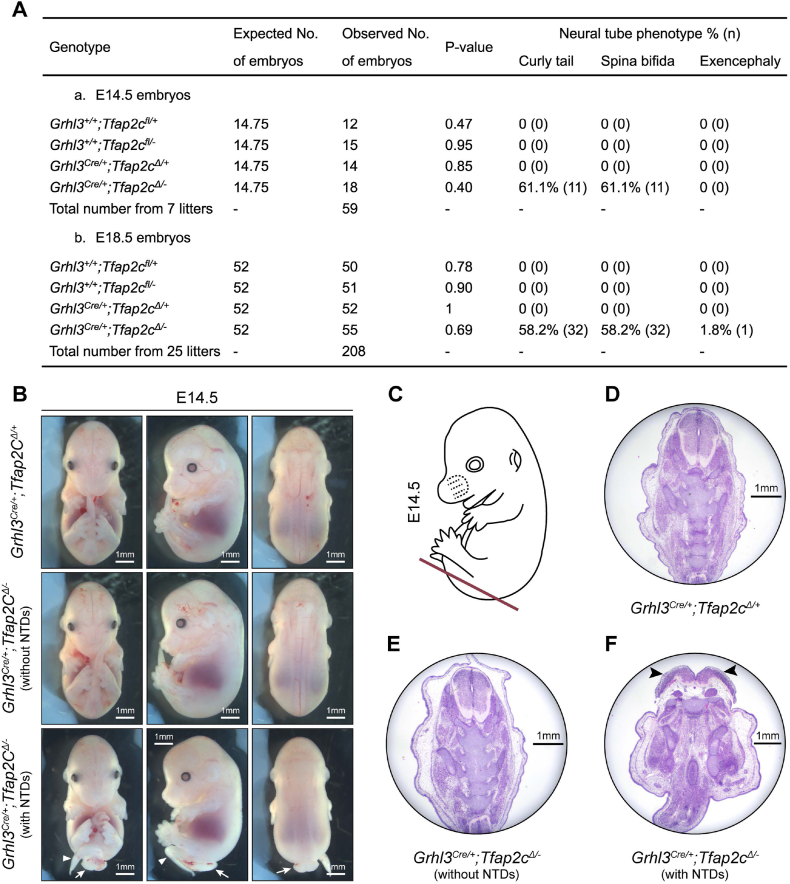
Specific inactivation of *Tfap2c* expression in *Grhl3*-expressing cells induces partially penetrate spinal neural tube defects with reduced severity. (A) Expected and observed numbers of embryos carrying corresponding genotypes from the cross of *Grhl3*^*Cre/+*^*;Tfap2c*^*+/−*^ and *Tfap2c*^*fl/fl*^ mice at E14.5 and E18.5, and the penetrance of neural tube defects in these embryos. The expected numbers of embryos were calculated based on the expected frequency of each genotype using the total number of collected embryos at each timepoint. A one sample χ2 test was used to compare the expected and observed number of embryos. (B) The gross appearances of E14.5 *Grhl3*^*Cre*^*;Tfap2c*^*flox*^ embryos. The *Grhl3*^*Cre/+*^*;Tfap2c*^*Δ/-*^ cKO embryos only displayed partially penetrate curly tail (white triangles) and low lumbo-sacral spina bifida (white arrows). (C) Schematic illustration showing the lower spinal position of transverse sectioning of E14.5 embryos in (D–E). (D) Transverse sectioning of Grhl3 Cre/+;Tfap2cΔ/+ control embryos at the lower spinal region showing a closed neural tube. (E) Transverse sectioning of *Grhl3*^*Cre/+*^*;Tfap2c*^*Δ/-*^ cKO embryos without neural tube defects at the lower spinal region showing a closed neural tube. (F) Transverse sectioning of *Grhl3*^*Cre/+*^*;Tfap2c*^*Δ/-*^ cKO embryos with neural tube defects at the lower spinal region showing a convex neural plate (black arrowheads).

As neither the *Tfap2a-Cre* nor the *Tfap2c-Cre* specifically confined deletion of *Grhl3* to the neural plate border cells, we pivoted in our approach and decided to target *Tfap2c* expression in these cells using a *Grhl3-Cre* driver. Our reasoning focused on the fact that *Grhl3* and *Tfap2c* expression overlaps in the minute neural plate border cell cohort at E8.5 and that disruption of either might induce NTD. To achieve this, we crossed the *Tfap2c*^*fl/fl*^ line to a *CMV-Cre* strain and then backcrossed the double heterozygous offspring to WT *C5*7BL*/6* mice to constitutively delete one allele of *Tfap2c* (Fig. 6C). *Grhl3*^*Cre/+*^ mice were then crossed to this *Tfap2c*^*+/−*^ line to generate double heterozygotes (*Grhl3*^*Cre/+*^*;Tfap2c*^*+/−*^). None of these animals exhibited NTD. The double heterozygous mice were crossed to the *Tfap2c*^*fl/fl*^ line to produce offspring that are either WT or heterozygous for *Grhl3-Cre* and carry one *Tfap2c* floxed allele with either a WT *Tfap2c* allele or a *Tfap2c*-null allele (Fig. 6A–E). When the *Grhl3-Cre* is expressed, the floxed *Tfap2c* exon 6 will be deleted in the *Grhl3*^*Cre/+*^*;Tfap2c*^*Δ/+*^ control and *Grhl3*^*Cre/+*^*;Tfap2c*^*Δ/-*^ cKO mice (Fig. 6A–E) [58]. Of note, and different from the *Tfap2a-* and *Tfap2c-Cre*, the *Cre* recombinase gene was inserted into the exon 1 of *Grhl3*, rendering the *Grhl3-Cre* allele a null allele (Fig. 6A) [9,30]. Examination of the pattern of *Grhl3-Cre*-mediated *Tfap2c* excision across three germ layers in the cKO embryos showed that a complete deletion of *Tfap2c* floxed region only occurred in the ectoderm-derived epidermis, while mesoderm-derived heart and endoderm-derived liver retained a large fraction of undeleted flox *Tfap2c* alleles (Fig. 6F). At both E14.5 and E18.5, embryos with all four genotypes (*Grhl3*^*+/+*^*;Tfap2c*^*fl/+*^, *Grhl3*^*+/+*^*;Tfap2c*^*fl/-*^, *Grhl3*^*Cre/+*^*;Tfap2c*^*Δ/+*^, and *Grhl3*^*Cre/+*^*;Tfap2c*^*Δ/-*^) were present in a normal Mendelian ratio, indicating that the conditional knock-out of *Tfap2c* did not cause embryonic lethality (Fig. 7A).

**Fig. 8:**
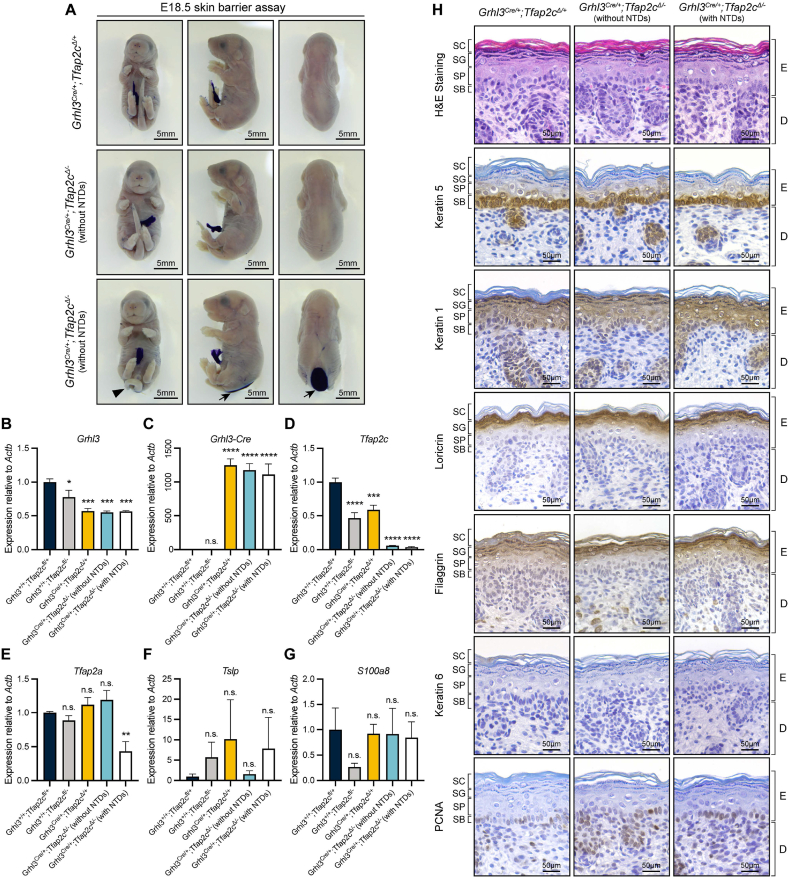
Specific inactivation of *Tfap2c* expression in *Grhl3*-expressing cells does not induce classical *Grhl3*-null phenotypes. (A) Skin barrier assay on E18.5 *Grhl3*^*Cre*^*;Tfap2c*^*flox*^ embryos showing a fully acquired epidermal barrier in both *Grhl3*^*Cre/+*^*;Tfap2c*^*Δ/+*^ control (N = 4) and *Grhl3*^*Cre/+*^*;Tfap2c*^*Δ/-*^ cKO animals (N = 5). Black triangles, curly tail; black arrows, spina bifida. (B–G) Q-RT-PCR analyses on E18.5 epidermis showing mRNA abundance of *Grhl3*, *Grhl3-Cre*, *Tfap2c*, *Tfap2a*, *Tslp* and *S100a8*. Bar graphs presented as mean ± standard error of mean. A one-way ANOVA test following by a Dunnett's multiple comparison test between *Grhl3*^*+/+*^*;Tfap2c*^*fl/+*^ embryos and other genotypes were used for data analysis. * = P-value <0.05, ** = P-value <0.01, *** = P-value <0.001, **** = P-value <0.0001. n.s., not significant. (H) Hematoxylin and eosin staining and immunohistology analysis of E18.5 *Grhl3*^*Cre/+*^*;Tfap2c*^*Δ/+*^ control and *Grhl3*^*Cre/+*^*;Tfap2c*^*Δ/-*^ cKO skin. N = 4. SC, stratum corneum; SG, stratum granulosum; SP, stratum spinosum; SB, stratum basale; E, epidermis; D, dermis.

Importantly, no NTDs were observed in the *Grhl3*^*Cre/+*^*;Tfap2c*^*Δ/+*^ control animals, while around 60 % of *Grhl3*^*Cre/+*^*;Tfap2c*^*Δ/-*^ cKO embryos had curly tail and spina bifida at both E14.5 and E18.5 (Fig. 7A). Interestingly, the spina bifida in these animals showed a reduced severity as evidenced by the low lumbo-sacral lesion (Fig. 7, Fig. 8A), whereas in *Grhl3*-null, *Grhl3*^*Δ/-*^*;Tfap2a*^*Cre/+*^ cKO and *Grhl3*^*Δ/-*^*;Tfap2c*^*Cre/+*^ cKO mice, the lesion normally extends rostrally into the thoracic region. This is consistent with the transverse sectioning at E14.5 showing a malformed convex neural plate at the low lumbo-sacral region of the *Grhl3*^*Cre/+*^*;Tfap2c*^*Δ/-*^ cKO embryos with caudal NTDs (Fig. 7C and F). The cKO mice without NTDs displayed a normal closed neural tube identical to the *Grhl3*^*Cre/+*^*;Tfap2a*^*Δ/+*^ control embryos (Fig. 7C–E). The full-body skeletal preparations on E18.5 *Grhl3*^*Cre/+*^*;Tfap2c*^*Δ/-*^ cKO embryos with NTDs showed that consistent with their gross appearance, the lesion of spina bifida was significantly lower compared with that of *Grhl3*-null, *Grhl3*^*Δ/-*^*;Tfap2a*^*Cre/+*^ cKO and *Grhl3*^*Δ/-*^*;Tfap2c*^*Cre/+*^ cKO mice, with the first SVP identified at T12 or T13 (Figs. S4D–E). Moreover, we did not observe a strong kyphosis phenotype in *Grhl3*^*Cre/+*^*;Tfap2c*^*Δ/-*^ cKO embryos with NTDs (Fig. S4D). The vertebral phenotype of the *Grhl3*^*Cre/+*^*;Tfap2c*^*Δ/-*^ cKO embryos without NTDs did not differ from the controls (Fig. S4D). These findings indicate that loss of *Tfap2c* expression in the *Grhl3*-expressing neural plate border cell population is sufficient to induce caudal NTDs with reduced severity and penetrance to those observed in *Grhl3*-null, *Grhl3*^*Δ/-*^*;Tfap2a*^*Cre/+*^ cKO and *Grhl3*^*Δ/-*^*;Tfap2c*^*Cre/+*^ cKO animals.

To investigate if *Grhl3*^*Cre/+*^*;Tfap2c*^*Δ/-*^ cKO embryos exhibited other *Grhl3*-null phenotypes, we harvested E18.5 embryos and examined general morphology and skin barrier function. These embryos exhibited a normal body shape and limb and digit morphology (Fig. 8A–S4D, and S5C). In addition, the *Grhl3*^*Cre/+*^*;Tfap2c*^*Δ/-*^ cKO embryos also exhibited a normal skin barrier development (Fig. 8A). H&E staining and IHC analyses showed a normal thickness and morphology of the *Grhl3*^*Cre/+*^*;Tfap2c*^*Δ/-*^ cKO epidermis at E18.5 without SC compaction and SG/SP expansion. The expression domains of K5, K1, loricrin, and filaggrin were all comparable to that of the *Grhl3*^*Cre/+*^*;Tfap2c*^*Δ/+*^ control skin (Fig. 8H). In addition, K6 expression was absent in the cKO skin and the PCNA expression was also confined to the basal layer with well-organised lining of PCNA-positive cells (Fig. 8H).

Real-time quantitative polymerase chain reaction (Q-RT-PCR) analyses showed that in E18.5 epidermis from mice heterozygous for *Grhl3-Cre*, *Grhl3* expression level was reduced to around 50 % of the *Grhl3*^*+/+*^*;Tfap2c*^*fl/-*^ control level due to the disruption of *Grhl3* exon 1 by the integration of the *Cre* recombinase gene (Fig. 8B). Also, *Tfap2c* mRNA abundance was reduced to a minimal level in the *Grhl3*^*Cre/+*^*;Tfap2c*^*Δ/-*^ cKO embryos regardless the presence of NTDs (Fig. 8D). Intriguingly, in the cKO embryos without NTDs, the expression level of *Tfap2a* did not differ from the controls, whereas it was significantly reduced in cKO mice with NTDs (Fig. 8E). This is likely due to the genetic interaction between *Tfap2a* and the partial loss of *Grhl3* due to the integration of *Cre* gene in the context of neurulation as shown previously [51]. In keeping with the normal epidermal barrier function and architecture, the expression of *Tslp* and *S100a8* was also comparable to the control level (Fig. 8F–G). As a core component of the PCP pathway in epidermal structures, GRHL3 is required to direct keratinocyte migration in wound healing and hence loss of GRHL3 leads to defective wound repair [10,12]. Therefore to confirm that keratinocyte migration is also normalised in the *Grhl3*^*Cre/+*^*;Tfap2c*^*Δ/-*^ cKO mice, we next performed an *in vitro* scratch assay on primary keratinocyte isolated from E18.5 epidermis. We found that the speed of wound closure did not differ between the *Grhl3*^*+/+*^*;Tfap2c*^*fl/+*^ control and *Grhl3*^*Cre/+*^*;Tfap2c*^*Δ/-*^ cKO mice regardless the presence of NTDs. Moreover, at 48 h post scratching, a complete wound closure was achieved in all groups, suggesting that loss of TFPA2C in *Grhl3*-expressing cells did not compromise epidermal migration in wound healing (Figs. S9A–B). Together, these results demonstrate a clear de-association between neural tube closure and other developmental events, specifically surface ectoderm development, hence, we conclude that the neural plate border cell cohort marked by *Tfap2c* and *Grhl3* expression is a crucial population for the early-stage spinal neurulation.

## 3 Discussion

In the current study, we utilised scRNA-seq to interrogate the transcriptomic profiles of the caudal half of the WT and *Grhl3*-null embryos at E8.5 (the 6-7-somite stage). We defined a unique cellular cohort characterised by expression of *Grhl3*, *Tfap2a*, and *Tfap2c* that is neither committed surface ectoderm nor neuroepithelial/neural crest cells, but a previously unidentified novel progenitor population. We subsequently showed that targeted loss of *Grhl3* expression in *Tfap2a*-expressing cells is sufficient to induce NTDs, and that loss of *Tfap2c* in *Grhl3*-expressing cells also induced spinal NTDs. Most importantly, through the latter model, we demonstrated a clear de-association between neural tube closure and surface ectoderm development as evidenced by the absence of epidermal abnormalities in the *Tfap2c-*cKO embryos with NTDs. Taken together, we conclude that the neural plate border cell population marked by *Grhl3*, *Tfap2a*, and *Tfap2c* expression plays a pivotal role in early-stage neurulation, in agreement with the findings from the tissue explant studies in chicken [[23], [24], [25]].

The cellular identity of *Grhl3*-expresssing neural plate border cell population at E8.5 has to date remained undefined. It was previously thought to represent committed surface ectoderm, given that GRHL3 is critical for epidermal development during later stages of gestation [7,9,12], and that in the cranial and anterior spinal regions, GRHL3 has been shown to mediate fate specification of surface ectoderm from the neural plate border territory [17]. Possible links between the *Grhl3*-expressing neural plate border cells and neural crest development have also been mooted, despite *Grhl3*-null animals failing to display abnormalities in neural crest development [7,9]. Through scRNA-seq, we showed that at E8.5, the caudal *Grhl3*-expressing neural plate border cell population represents a previously undefined novel progenitor population that lacks a neural crest fate. This is despite co-expression of *Grhl3* with *Tfap2a* and *Tfap2c*, both previously characterised markers associated with neural crest specification [37,[47], [48], [49]].

Previous studies have explored the role of *Grhl3* expression in different tissues in neural tube closure. De Castro et al. [19] demonstrated that specific inactivation of *Grhl3* expression in hindgut using *Sox17-Cre* can lead to mild caudal NTDs, with an incomplete penetrance. However, at the early stage of spinal neurulation, the timepoint when the closure defect starts to be evident [7,19], *Grhl3* expression is not detectable in the gut endoderm [13,19]. Hence, an additional site must be required to fulfil the requirement of *Grhl3*-sepcific function in early-stage neural plate bending. Here, based on the finding from our scRNA-seq, we targeted the *Grhl3*-expressing population located within the neural plate border domain at E8.5 and demonstrated the direct inductive role of this cell cohort in early spinal neurulation through our *Grhl3*^*Cre/+*^*;Tfap2c*^*Δ/-*^ cKO model. This is in keeping with the previous tissue explant studies in chicken showing the dispensable role of surface ectoderm in neural tube closure, and the finding that at both E9.5 and E10.5, loss of *Grhl3* expression does not compromise the morphology and integrity of surface ectoderm along the entire spinal region encompassing both closed and open neural tube [21,[23], [24], [25]]. Together, De Castro et al. [19] and our data indicate that spinal neural tube closure requires collaborative efforts from at least two *Grhl3*-expressing sites: the initiation and early progression of spinal neurulation is dependent on the cell cohort marked by *Grhl3*, *Tfap2a*, and *Tfap2c* expression, while the hindgut-specific *Grhl3* function is indispensable for mid-to late-stage spinal neural tube morphogenesis. The priority for the future works is to uncover the *Grhl3*-dependent molecular pathways in these neural plate border cells that induce neural tube closure. The convex caudal neural plate in both *Grhl3*^*Δ/-*^*;Tfap2a*^*Cre/+*^ cKO and *Grhl3*^*Cre/+*^*;Tfap2c*^*Δ/-*^ cKO animals at E14.5 suggests that the neural plate border cell cohort marked by *Grhl3*, *Tfap2a*, and *Tfap2c* expression may guide apical constriction and cell shape change within neuroepithelium to generate necessary force that induces neural plate juxtaposition; secondly, neural plate border GRHL3 is highly likely to be required to establish a robust cellular protrusions network to bridge the bilateral neural folds while ‘zippering’ progresses caudally as *Grhl3*-null animals show altered protrusion dynamics along the neural plate border [21]. Hence, future research could focus on investigating the GRHL3-dependent regulatory networks involved in neural plate juxtaposition and in generating or safeguarding cellular projection networks at the caudal neural plate border during neurulation.

Another important finding from the current study is the clear de-association between neural crest development and neural tube closure. We showed that in the *Grhl3*^*Cre/+*^*;Tfap2c*^*Δ/-*^ cKO mice, abolition of *Tfap2c*, the critical gene for early neural crest induction from the neural plate border territory,^48 37^ specifically in *Grhl3*-expressing neural plate border cells, did not lead to any classic phenotypes caused by abnormal neural crest development. This indicates that the small cohort of neural plate border cell population marked by *Grhl3*, *Tfap2a*, and *Tfap2c* expression does not contribute to neural crest population although these cells express the markers of neural crest progenitor and early neural crest genes and reside within the neural plate border territory. In addition, this finding strongly supports the view that neural plate border is a region with molecular heterogeneity and great flexibility in cell fates [49,59]. Therefore, we postulate that neural plate border GRHL3 is a critical factor to distinguish the non-neural crest lineage that is essential for spinal neurulation. The presence of a GRHL3-dependent regulatory network in the naïve neural plate border cells may be able to supress the neural crest fate decision mediated by TFAP2A and TFAP2C and in turn promote the specification of the novel progenitor population crucial for neural tube closure from the neural plate border territory. This finding again provides an excellent example for the multipotent property of neural plate border and new insights in understanding the complex molecular architecture that medicates neural plate border segregation.

In addition to these findings, the scRNA-seq dataset we generated in the current study offers valuable insights with significant implications for future studies. For example, we noticed that the E8.5 *Grhl3*-null embryos showed a significant loss of mesoderm-specific populations, including pre-somitic mesoderm, somitic mesoderm, lateral plate mesoderm, and caudal mesoderm (Fig. 1A). In late gestation, the null animals show defective axial elongation with a short longitudinal body axis, which was believed to be caused by defective convergent extension due to disruption in PCP pathway [10]. In addition, the *Grhl3*-null animals also show a severe kyphosis at the lower spinal region [7]. Here, our finding suggest that these abnormalities may also be the resulting outcome of a defective mesoderm development in the absence of GRHL3. Intriguingly, however, we did not see a significant abundance change in the paraxial mesoderm population (Cluster 1, Fig. 1A). This is very surprising as the precursor of paraxial mesoderm, the pre-somitic mesoderm cluster, was almost absent in our scRNA-seq analysis. However, *Grhl3*-null mice do not develop any defects in somite formation and segmentation [7,9]. During early embryogenesis, both lateral and paraxial mesoderm progenitors (LPMPs) from CLE region and NMPs from NSB region contribute to the pre-somitic mesoderm population [60], the anterior proportion of the pre-somitic mesoderm becomes paraxial mesoderm which then undergoes somitogenesis [61]. Therefore, a loss of pre-somitic mesoderm without a significant reduction in paraxial mesoderm population is perplexing. One possible explanation is that both pre-somitic and somitic mesoderm populations may have been exhausted due to a premature differentiation into paraxial mesoderm, but this is not consistent with the absence of defects in somite formation and segmentation in *Grhl3*-null embryos. Previous research has shown that the progenitor populations for pre-somitic mesoderm, LPMPs and NMPs, emerge from CLE and NSB regions at around E7.5, respectively [60]. In keeping with this is the observation that *Grhl3* is expressed at low levels in these two regions at E8.5,^19^ therefore it is reasonable to speculate that *Grhl3* expression at E8.5 in CLE and NSB regions is required for promoting pre-somitic mesoderm commitment from NMPs and pre-somitic, lateral plate and caudal mesoderm differentiation from LPMPs, whereas in *Grhl3*-null embryos, the initial mesoderm specification from these progenitors and the development of paraxial mesoderm is normal, until E8.5 when the production of pre-somitic, lateral plate and caudal mesoderm cells is largely stalled due to loss of GRHL3. This hypothesis is also supported by the loss of progenitors for mesoderm populations as seen in the RNA velocity result (Fig. S2A). Whether GRHL3 is required for LPMPs and NMPs differentiation remains an avenue for further research.

One limitation of the current study is that our scRNA-seq analysis involved only a single timepoint in development, providing a static snapshot of the transcriptomic landscape at the initiation stage of neurulation. To capture the dynamic transcriptomic changes throughout neural tube morphogenesis, future studies should perform scRNA-seq or spatial transcriptomic analysis at multiple developmental timepoints. This approach would characterise the full spectrum of gene expression changes across the entire neurulation stage in WT and *Grhl3*-null animals. It could also help to further investigate the molecular mechanisms by which the *Grhl3*-expressing neural plate border cell cohort regulates neurulation and neural plate border segregation along the trajectory of early embryonic development. Additionally, future lineage tracing studies will be necessary to validate the lineage fates of the predicted progenitor population from our RNA velocity and Monocle 3 analyses.

In conclusion, our findings from the current study underscore the significance of the unique neural plate border cell population marked by *Grhl3*, *Tfap2a*, and *Tfap2c* expression in neural tube closure and demonstrated that early spinal neurulation is independent of neural crest and epidermal development. This is a major step forward in the understanding of the mechanism underlying neural tube closure at the cellular level and provides us with an excellent platform to further investigate *Grhl3*-dependent pathways that induce neural tube closure.

## Data availability statement

The single cell-RNA sequencing dataset generated by this study has been deposited into Gene Expression Omnibus (GEO) repository under the accession number GSE246181. All dataset analyses were performed based on R (version 3.6.0) [62]. This paper does not report original code. Any additional information required for re-analysing the datasets reported in this paper is available from the corresponding authors upon request.

## Materials and methods

### Mice

To conditionally inactivate *Grhl3* expression in *Tfap2a*-expressing cells, a line heterozygous for *Grhl3* and *Tfap2a-Cre* (*Grhl3*^*+/−*^*;Tfap2a*^*Cre/+*^) was generated by crossing *Grhl3*^*+/−*^ mice and a *Tfap2a*^*Cre/Cre*^ line (Fig. 3A). The *Tfap2a-Cre* allele contains an Internal Ribosome Entry Site (*IRES*)-*Cre* cassette which is inserted into the endogenous *Tfap2a* 3’ untranslated region (UTR). This line was obtained from Professor Anne Moon from the University of Utah. The *Grhl3*^*+/−*^*;Tfap2a*^*Cre/+*^ mice were then crossed to the *Grhl3*^*fl/fl*^ line through timed mating to generate *Tfap2a*-*Cre*-heterozygous or WT *Tfap2a*-homozygous offspring with one floxed *Grhl3* allele and either a WT *Grhl3* allele or a *Grhl3*-null allele (Fig. 3C). The floxed *Grhl3* region will be excised, rendering a *Grhl3* delta allele, when the *Tfap2a*-*Cre* is expressed (Fig. 3B). The generation of the *Grhl3*^*+/−*^ line [7], *Grhl3*^*fl/fl*^ line [56], and *Tfap2a*^*Cre/Cre*^ line [54] has been described previously.

To achieve specific inactivation of *Grhl3* expression in *Tfap2c*-expressing cells, a *Tfap2c*^*Cre/+*^ mouse strain was created through the Monash Genome Modification Platform. Briefly, a cassette containing a self-cleaving porcine teschovirus-1 2A (P2A) peptide sequence and a *Cre* recombinase gene was knocked into the endogenous *Tfap2c* locus preceded to the *Tfap2c* stop codon through CRISPR-Cas9 system with a ssDNA repair template (Fig. S6A). The *Tfap2c*^*Cre/+*^ strain was then crossed to the *Grhl3*^*+/−*^ mice to generate the double heterozygous *Grhl3*^*+/−*^*;Tfap2c*^*Cre/+*^ line, which was then timed mated with the *Grhl3*^*fl/fl*^ mice to generate offspring that carry one *Tfap2c*-*Cre* allele, one floxed *Grhl3* allele and one *Grhl3*-null allele (Fig. S6C). The presence of the *Cre* allele will inactivate the floxed *Grhl3* allele in *Tfap2c*-expressing cells (Fig. S6B).

To delete TFAP2C in *Grhl3*-expressing cells, a *Tfap2c*^*fl/fl*^ line was obtained from Professor Trevor Williams from the University of Colorado (Fig. 6B). The *Tfap2c*^*fl/fl*^ mice were firstly crossed to a *CMV-Cre* strain to generate double heterozygotes. The double heterozygous offspring were then backcrossed to WT *C5*7BL*/6* mice to generate the *Tfap2c*^*+/−*^ line, which was further crossed to *Grhl3*^*Cre/+*^ mice to generate *Grhl3*^*Cre/+*^*;Tfap2c*^*+/−*^ mice. Finally, the double heterozygous *Grhl3*^*Cre/+*^*;Tfap2c*^*+/−*^ mice were crossed to the *Tfap2c*^*fl/fl*^ line to generate the *Grhl3*^*Cre/+*^*;Tfap2c*^*Δ/-*^ cKO embryos (Fig. 6C). In the cKO mice, the floxed *Tfap2c* exon 6 will be excised by the *Grhl3-Cre* (Fig. 6B). The generation of the *Grhl3*^*Cre/+*^ line [30] and *Tfap2c*^*fl/fl*^ line [58] has been described previously.

To obtain *Grhl3*^*−/−*^ embryos, the *Grhl3*^*+/−*^ mice were intercrossed through timed mating overnight. All experimental mouse lines described above were maintained on a pure C57BL/6 background and raised under optimised conditions with standard food and water supplied *ad libitum*. For timed mating, male and female mice (generally older then 8 weeks) that have reached sexual maturity were placed in the same cage overnight. The female mice were then inspected for a vaginal plug the morning following the mating. The gestational age of embryos was identified as E0.5 at mid-day on the day of detection of a vaginal plug. To harvest embryos, pregnant female mice were sacrificed through slow-fill CO_2_ asphyxiation following by cervical dislocation. For experiments that require an intact whole-body structure or intact skin integrity, the hypothermia method was used to euthanise embryos aged > E10.0. For other experiments, embryos aged > E10.0 were fully euthanised by decapitation. Mouse genotypes or deletion of the floxed alleles were all confirmed by conventional PCR using genomic DNA isolated from yolk sac samples or different tissues and organs with GoTaq Master Mixes (Promega) and oligonucleotide primer sets listed in Table S2. All animal experiments were pre-approved by the Alfred Research Alliance Animal Ethics Committee with project numbers E/1800/2018/M, E/1900/2019/M, E/8285/2022/M, and E/8286/2022/M. All studies were conducted in the accordance of the Australian Code for the Care and Use of Animals for Scientific Purposes and the Australian Code for the Responsible Conduct of Research and in compliance with the ARRIVE guidelines.

### Embryonic tissue dissociation for scRNA-seq

To generate animals for scRNA-seq, *Grhl3*^*+/−*^ mice were intercrossed through timed mating. E8.5 (the 6-7-somite stage) embryos were harvested at 3 p.m. on the eighth day post-fertilisation. Embryos were dissected in DEPC-PBS and the yolk sac samples were utilised for genotyping as described above. The caudal half of five WT and three *Grhl3*^*−/−*^ embryos was separated using tungsten needles in 0.04 % BSA (Sigma-Aldrich) in DEPC-PBS and then pelleted through centrifugation at 300 rcf for 3 min at 4 °C (Fig. 1A). The supernatant was removed, and the caudal half of the embryo was then incubated with 50 μL 0.25 % (v/v) trypsin (Gibco) in DEPC-PBS for 5 min at 37 °C and triturated with a 200 μL pipette tip to dissociate tissues. The digestion reaction was then inactivated by adding 100 μL 10 % (v/v) FBS (Bovogen Biologicals) in DMEM (Gibco). Cells from five WT embryos and from three *Grhl3*^*−/−*^ embryos were pooled and filtered through Flowmi Cell Strainers (40 μm). Cells were then pelleted through centrifugation at 300 rcf for 3 min at 4 °C and resuspended in 15 μL 10 % (v/v) FBS (Bovogen Biologicals) in DMEM (Gibco) per embryo. Cell number and viability were assessed using a hemocytometer by mixing 8 μl cell suspension with 8 μl 0.4 % (v/v) Trypan Blue Solution (Sigma-Aldrich) and a ∼95 % cell viability was observed in both WT and *Grhl3*^*−/−*^ samples. Cells were then diluted in 10 % (v/v) FBS (Bovogen Biologicals) in DMEM (Gibco) to a final concentration of 1000 cells/μL for single cell droplet generation and library preparation (Fig. 1A).

### ScRNA-seq library preparation and sequencing

A Chromium Next GEM Single Cell 3′ GEM, Library & Gel Bead Kit v3.1 and a Chromium Controller from 10X Genomics were used to generate single cell droplets as per manufacturer's instructions. Two WT and two *Grhl3*^*−/−*^ reactions were performed. Briefly, cell suspension with a master mix, gel beads and partitioning oil were loaded onto a Chromium Chip B to generate Gel Beads-in-emulsion (Fig. 1A). After this, samples were transferred into PCR tube strips and processed for reverse transcription and template switching using a T100 Thermal Cycler (Bio-Rad Laboratories). Synthesised full-length cDNA was then purified and amplified, followed by a clean-up reaction. The quality and quantity of amplified cDNA were assessed using an Agilent 4200 TapeStation with a High Sensitivity D5000 ScreenTape. Two WT and two *Grhl3*^*−/−*^ 3′ gene expression libraries were then constructed for sequencing as outlined in the manufacturer's user guide. In short, cDNA amplicon from the previous reaction was fragmented and ligated with adaptors, and then subjected for a sample index PCR and a size selection with SPRIselect reagent. The quality and quantity of the libraries were again assessed using an Agilent 4200 TapeStation with a High Sensitivity D5000 ScreenTape. Finally, the libraries were sequenced on an Illumina NextSeq 500 System with the NextSeq 500/550 High Output Kit v2.5 at a targeted sequencing depth of 400 million reads per library (Fig. 1A).

### ScRNA-seq data computational analysis

The raw sequencing data were processed using Cell Ranger (10X Genomics, version 3.0.0) with MM10 referenced genome. The raw dataset contains 24,239 cells with the post-normalisation mean reads per cell at 74,836 and the median genes per cell at 5537. Then, cells were filtered using Seurat (version 3.0.2) [63]. Both WT and *Grhl3*^*−/−*^ cells with less than 3000 genes per cell were removed. Also, cells with less than 7500 read counts per cells and more than 7 % mitochondrial gene reads were removed from the dataset. A total of 20,196 cells passed the filtering and were retained in the final dataset. Then, all four libraries were merged into a single Seurat object and processed for SCTransform normalisation and dimension reduction. All cells were visualised using UMAP and unsupervised clustering was then performed using FindClusters function at the resolution of 0.4 and the marker genes of each cluster were identified using FindAllMarkers function. The published scRNA-seq reference atlas from Pijuan-Sala et al. [27] was used to manually annotate each cluster based on the cluster-specific marker genes.

For gene co-expression correlation analysis on *Grhl3* in cluster 10 only, the WT Cluster 10 was isolated, and the count matrix was extracted. The limma-voom pipeline (version 3.40.2) was then used to identify a set of co-expression genes whose expression changes were highly associated with *Grhl3* expression level in Cluster 10, with the log fold-change threshold of 0.45 [64]. The Pearson correlation coefficient tests were then performed on the set of genes with a 95 % confidence interval and the p-value of correlation smaller than 0.05. The result was visualised using the corrplot package (version 0.89) [65].

The RNA velocity analysis was performed using the Velocyto package [52]. The run10x function from Velocyto was used to generate the loom file, which contains the spliced, un-spliced and ambiguous read counts of each gene, based on the Cell Ranger output. The loom file was then loaded and converted into a single Seurat object using the SeuratWrappers package. Low quality cells were filtered as described above. Both global and cluster-specific RNA velocities were calculated using the RunVelocity function with the estimated nearest pooling reads across 25 neighbouring cells (kcells = 25). For the velocity of a single gene, the kcells values was set to 10. All velocity results were visualised using UMAP. The pseudotime trajectory analysis was performed using Monocle 3 [53]. First, the single-cell expression matrix was pre-processed and normalised. Then, batch effects between the samples were corrected using the “align_cds” function, and clustering was performed. The surface ectoderm and neural plate border cluster, marked by the co-expression of *Grhl3*, *Tfap2a*, and *Tfap2c* was selected. Pseudotime trajectories of the selected cluster were inferred separately for WT and *Grhl3*^*−/−*^ samples. The root of the modelled trajectory was identified as the node with the co-expression of *Grhl3*, *Tfap2a*, and *Tfap2c* in WT samples, and the corresponding node in *Grhl3*^*−/−*^ samples.

### Riboprobe construction and whole-mount *in situ* hybridisation

Riboprobe construction and whole-mount *in situ* hybridisation were performed as described previously [66]. All primer design templates and oligonucleotide primer sequences used for riboprobe template amplification are listed in Table S2. Whole-mount images of embryos were obtained using a Nikon SMZ1500 Stereomicroscope with the AxioVision software (Zeiss). Multiple images were taken and stacked using Adobe Photoshop to fully capture the pattern of staining. After imaging, embryos were fixed and embedded in paraffin as described previously [66], sectioned at 10 μm thickness in a transverse orientation and collected onto SuperFrost plus slides (Thermo Fisher Scientific), and processed for nuclear fast red staining using standard method. Slides were imaged using a Nikon ECLIPSE Ci-L upright microscope with the NIS-Elements D software.

### Histology and immunohistochemistry

For histological analyses, E14.5 whole embryos and E18.5 skin samples were fixed in 4 % (w/v) PFA in PBS at 4 °C for two to four days. All samples were processed using a Leica ASP300S Fully Enclosed Tissue Processor by Monash Histology Platform and embedded into paraffin. All samples were sectioned in a transverse orientation at 5 μm thickness, collected onto SuperFrost plus slides (Thermo Fisher Scientific) and air-dried for at least 24 h. H&E staining was performed as per standard protocol. Immunohistochemistry IHC analyses were performed using standard DAB methods with the ABC HRP Kit and the DAB Peroxidase Substrate Kit (Vector Laboratories). For IHC with mouse antibody, the Mouse-on-Mouse Immunodetection Kit (Vector Laboratories) was used as per the manufacturer's protocol. All sections were imaged using a Nikon ECLIPSE Ci-L upright microscope with the NIS-Elements D software. All antibodies and experimental conditions used for IHC are listed in Table S3.

### Skin barrier assay and skeletal preparation

For the skin barrier assay, E18.5 embryos were harvested and euthanise as above, then fixed in 100 % methanol on a roller mixer for 5 min and washed in PBS for 5 min twice. Fixed embryos were incubated in 0.1 % (w/v) toluidine blue (Sigma-Aldrich) in H_2_O for 5 min on a roller mixer, washed in PBS for 5 min twice and then imaged using a Canon EOS 60D camera. Skeletal preparations of E18.5 embryos were performed as described previously [67] and imaged using a Nikon SMZ1500 Stereomicroscope with the AxioVision software (Zeiss).

### Reverse transcription and quantitative polymerase chain reaction

E18.5 epidermis samples were separated from the dorsal skin by incubating in 1 mg/mL Dispase II powder (Gibco) in PBS at 4 °C for overnight. For RNA isolation, samples were homogenised in TRIsure (Bioline) using standard syringe method and RNA was then isolated according to the manufacturer's instructions. Then, RNA was subjected to DNase treatment using a TURBO DNA-free Kit (Invitrogen) to remove genomic DNA contamination and reverse transcribed using a Transcriptor First Strand cDNA Synthesis Kit (Roche) as per manufacturer's protocols. Q-RT-PCR was performed using the GoTaq qPCR Master Mix (Promega) with 10 ng cDNA in each reaction on a LightCycler 480 Instrument (Roche). Relative expression values were calculated using the ΔΔCT method by normalising genes of interest to *Actb* and analysed using Microsoft Excel and GraphPad Prism 10. All oligonucleotide primers used for Q-RT-PCR are listed in Table S2.

### Primary keratinocytes isolation and culturing

The isolation and culturing of mouse embryonic primary keratinocytes has been described previously [68]. Briefly, dorsal skin biopsies were obtained from E18.5 embryos and incubated in 4 mg/mL Dispase II powder (Gibco) in EpiLife Medium (Gibco) supplemented with 60 μM calcium and EpiLife Defined Growth Supplement (Gibco) overnight at 4 °C on a rotator. The epidermis was then separated from the underlying tissues and incubated in TrypLE Express Enzyme (Gibco) for 20 min at room temperature with gentle agitation. The primary keratinocytes were isolated by adding EpiLife Medium (Gibco) supplemented with 60 μM calcium and EpiLife Defined Growth Supplement (Gibco) to the digestion reaction and rubbing the epidermis vigorously using forceps. Isolated keratinocytes were then filtered through 100 μm cell strainers, centrifuged at 180 rcf for 5 min at 4 °C and resuspended in EpiLife Medium. Prior to seeding, culture plate was coated with Attachment Factor Protein (Gibco) for 30 min at 37 °C. Cells were seeded at the density of 5 × 10^4^/cm^2^ and incubated in a humidified incubator with 5 % CO_2_ at 37 °C. Culture medium was changed every 24 h to remove dead cells and spontaneously differentiated cells.

### Primary keratinocytes *in vitro* scratch assay

After a confluent monolayer of primary keratinocytes was formed, a sterile 200 μL pipette tip was used to scratch the monolayer. The culture medium was then immediately removed and replaced with fresh EpiLife Medium (Gibco) supplemented with 60 μM calcium and EpiLife Defined Growth Supplement (Gibco). Images of wounds at 0 h, 24 h and 48 h post-scratching were obtained using a Nikon Eclipse TS100 Inverted Phase Contrast Microscope and the extent of wound closure was quantified using Fiji [69] and GraphPad Prism 10.

### Statistical analysis

A one sample χ2 test was used to compare the expected and observed numbers of embryos at both E14.5 and E18.5, all P-values were determined based on one degree of freedom. Relative expression levels of genes, the thoracic vertebrae positions of the first SVP, body axis measurements, and the percentages of wound closure were compared between WT or control and other genotypes using one-way ANOVA tests following by Dunnett's multiple comparison tests. In Figs. S5A–B, the cranial-caudal axis lengths were also compared between two specific genotypes using Mann-Whitney tests. All statistical analyses were performed using GraphPad Prism 10.

## CRediT authorship contribution statement

**Zihao Deng:** Writing – review & editing, Writing – original draft, Visualization, Software, Project administration, Methodology, Investigation, Formal analysis, Data curation, Conceptualization. **Marina R. Carpinelli:** Supervision, Software, Project administration, Methodology, Investigation, Formal analysis, Data curation, Conceptualization. **Tariq Butt:** Project administration, Investigation. **Graham W. Magor:** Software, Methodology, Investigation, Formal analysis, Data curation. **Peinan Zhao:** Visualization, Software, Methodology, Investigation, Formal analysis, Data curation. **Kevin R. Gillinder:** Software, Formal analysis, Data curation. **Andrew C. Perkins:** Supervision, Software, Methodology, Investigation, Formal analysis, Data curation. **Stephen M. Jane:** Writing – review & editing, Writing – original draft, Supervision, Resources, Methodology, Investigation, Funding acquisition, Conceptualization.

## Declaration of competing interest

The authors declare that they have no known competing financial interests or personal relationships that could have appeared to influence the work reported in this paper.
