# Supplementary material for: Single-cell transcriptomics reveals the cellular identity of a novel progenitor population crucial for murine neural tube closure
Source: Heliyon. 2024 Aug 30;10(17):e37259. doi: 10.1016/j.heliyon.2024.e37259 (PMC11408003; doi:10.1016/j.heliyon.2024.e37259)
Supplement: Multimedia component 4 [file mmc4.pdf]

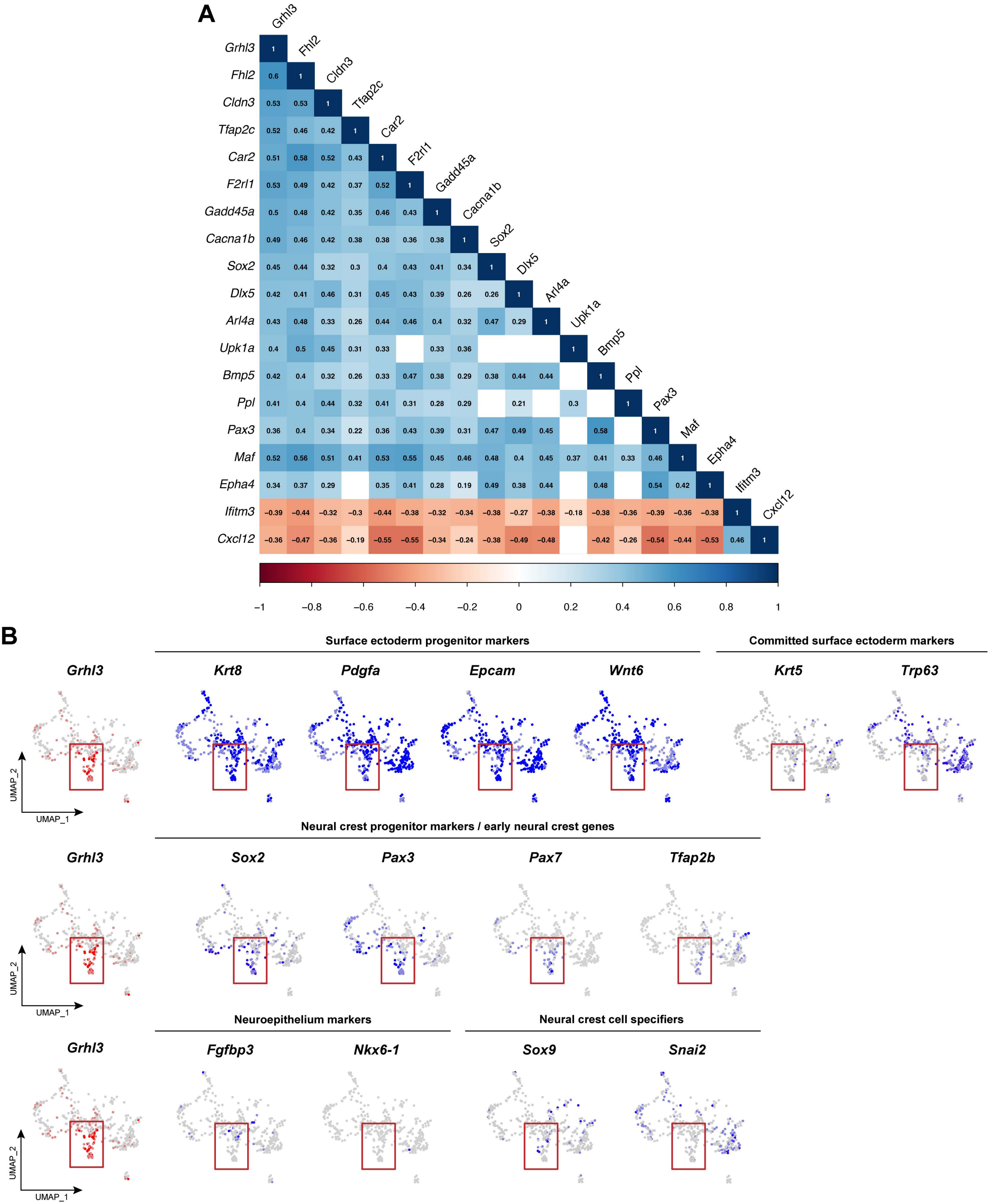
A
Grhl3
Fhl2
Cldn3
Tfap2c
Car2
F2rl1
Gadd45a
Cacna1b
Sox2
Dlx5
Arl4a
Upk1a
Bmp5
Ppl
Pax3
Maf
Epha4
Ifitm3
Cxcl12
−1
−0.8
−0.6
−0.4
−0.2
0
0.2
0.4
0.6
0.8
1
B
Surface ectoderm progenitor markers
Committed surface ectoderm markers
Grhl3
Krt8
Pdgfa
Epcam
Wnt6
Krt5
Trp63
UMAP_2
UMAP_1
Neural crest progenitor markers / early neural crest genes
Grhl3
Sox2
Pax3
Pax7
Tfap2b
Neuroepithelium markers
Neural crest cell specifiers
Grhl3
Fgfbp3
Nkx6-1
Sox9
Snai2

Figure S1

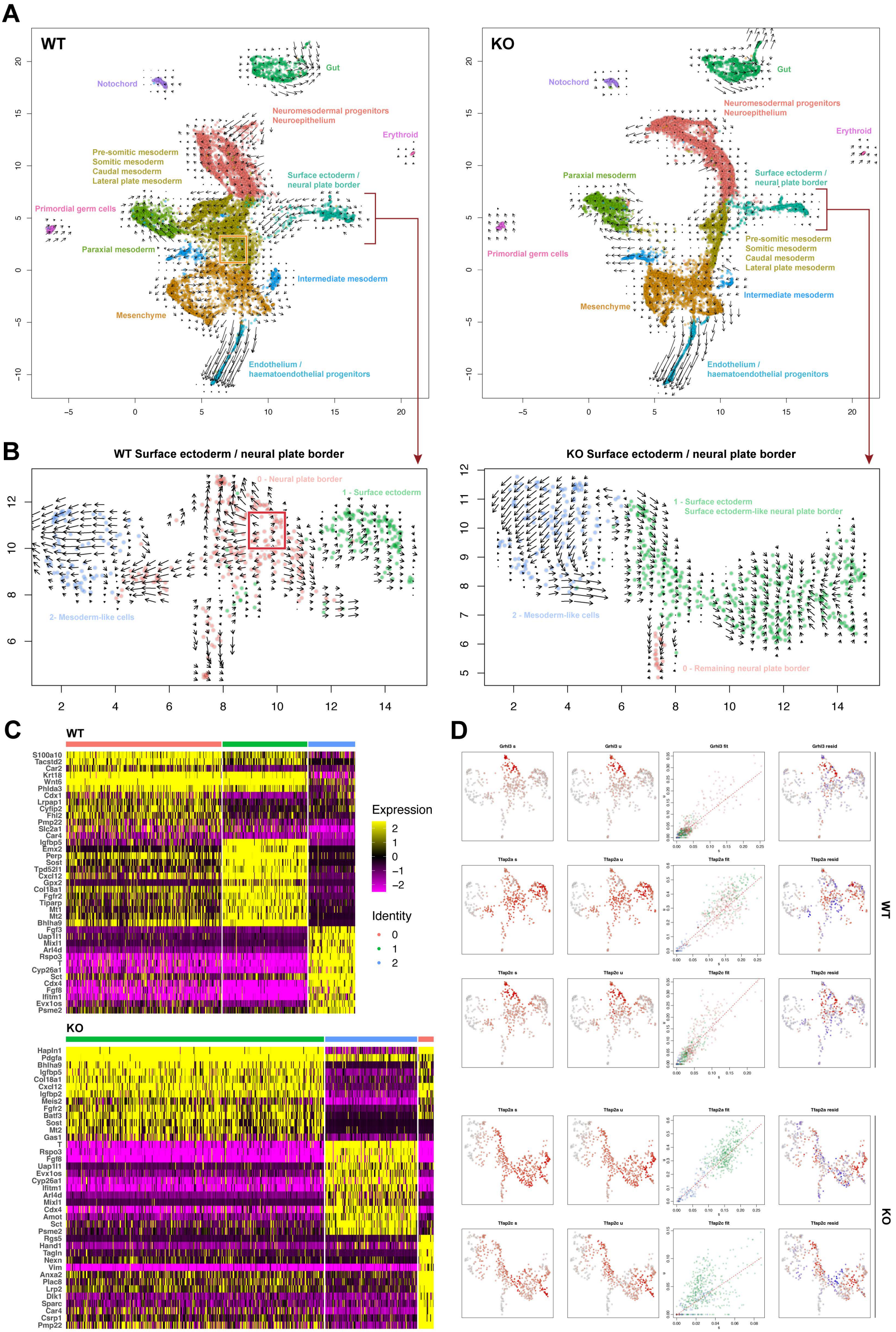
A
WT
Notochord
Gut
Neuromesodermal progenitors
Neuroepithelium
Erythroid
Pre-somitic mesoderm
Somitic mesoderm
Caudal mesoderm
Lateral plate mesoderm
Surface ectoderm / neural plate border
Primordial germ cells
Paraxial mesoderm
Intermediate mesoderm
Mesenchyme
Endothelium / haematoendothelial progenitors
KO
B
WT Surface ectoderm / neural plate border
0 - Neural plate border
1 - Surface ectoderm
2- Mesoderm-like cells
KO Surface ectoderm / neural plate border
1 - Surface ectoderm
Surface ectoderm-like neural plate border
2 - Mesoderm-like cells
0 - Remaining neural plate border
C
WT
S100a10
Tacstd2
Car2
Krt18
Wnt6
Phlda3
Cdx1
Lrpap1
Cyfip2
Fhl2
Pmp22
Slc2a1
Car4
Igfbp5
Emx2
Perp
Sost
Tpd52l1
Cxcl12
Gpx2
Col18a1
Fgfr2
Tiparp
Mt1
Mt2
Bhlha9
Fgf3
Uap1l1
Mixl1
Arl4d
Rspo3
T
Cyp26a1
Sct
Cdx4
Fgf8
Ifitm1
Evx1os
Psme2
Expression
Identity
KO
Hapln1
Pdgfa
Bhlha9
Igfbp5
Col18a1
Cxcl12
Igfbp2
Meis2
Fgfr2
Batf3
Sost
Mt2
Gas1
T
Rspo3
Fgf8
Uap1l1
Evx1os
Cyp26a1
Ifitm1
Arl4d
Mixl1
Cdx4
Amot
Sct
Psme2
Rgs5
Hand1
Tagln
Nexn
Vim
Anxa2
Plac8
Lrp2
Dlk1
Sparc
Car4
Csrp1
Pmp22
D
Grhl3 s
Grhl3 u
Grhl3 fit
Grhl3 resid
Tfap2a s
Tfap2a u
Tfap2a fit
Tfap2a resid
Tfap2c s
Tfap2c u
Tfap2c fit
Tfap2c resid
WT
KO

Figure S2

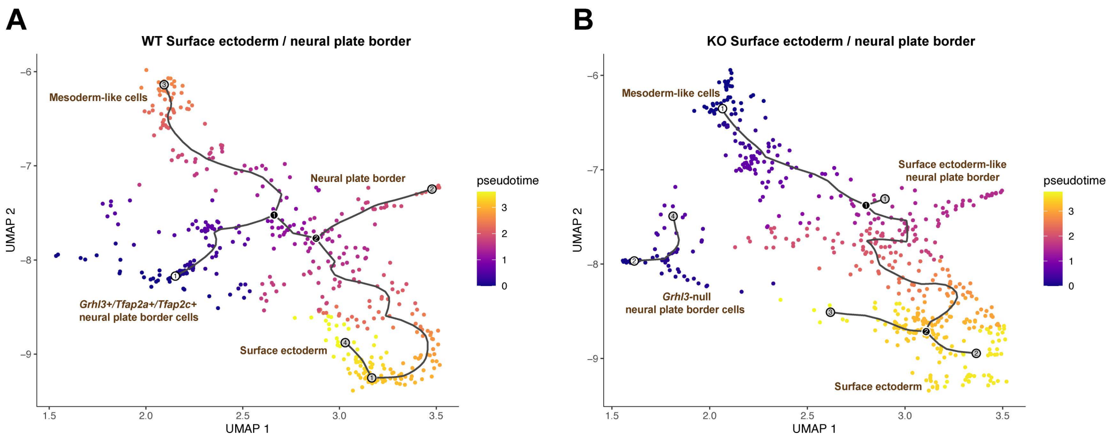
A
WT Surface ectoderm / neural plate border
Mesoderm-like cells
Neural plate border
Grhl3+/Tfap2a+/Tfap2c+
neural plate border cells
Surface ectoderm
UMAP 1
UMAP 2
pseudotime
B
KO Surface ectoderm / neural plate border
Mesoderm-like cells
Surface ectoderm-like
neural plate border
Grhl3-null
neural plate border cells
Surface ectoderm
UMAP 1
UMAP 2
pseudotime

Figure S3

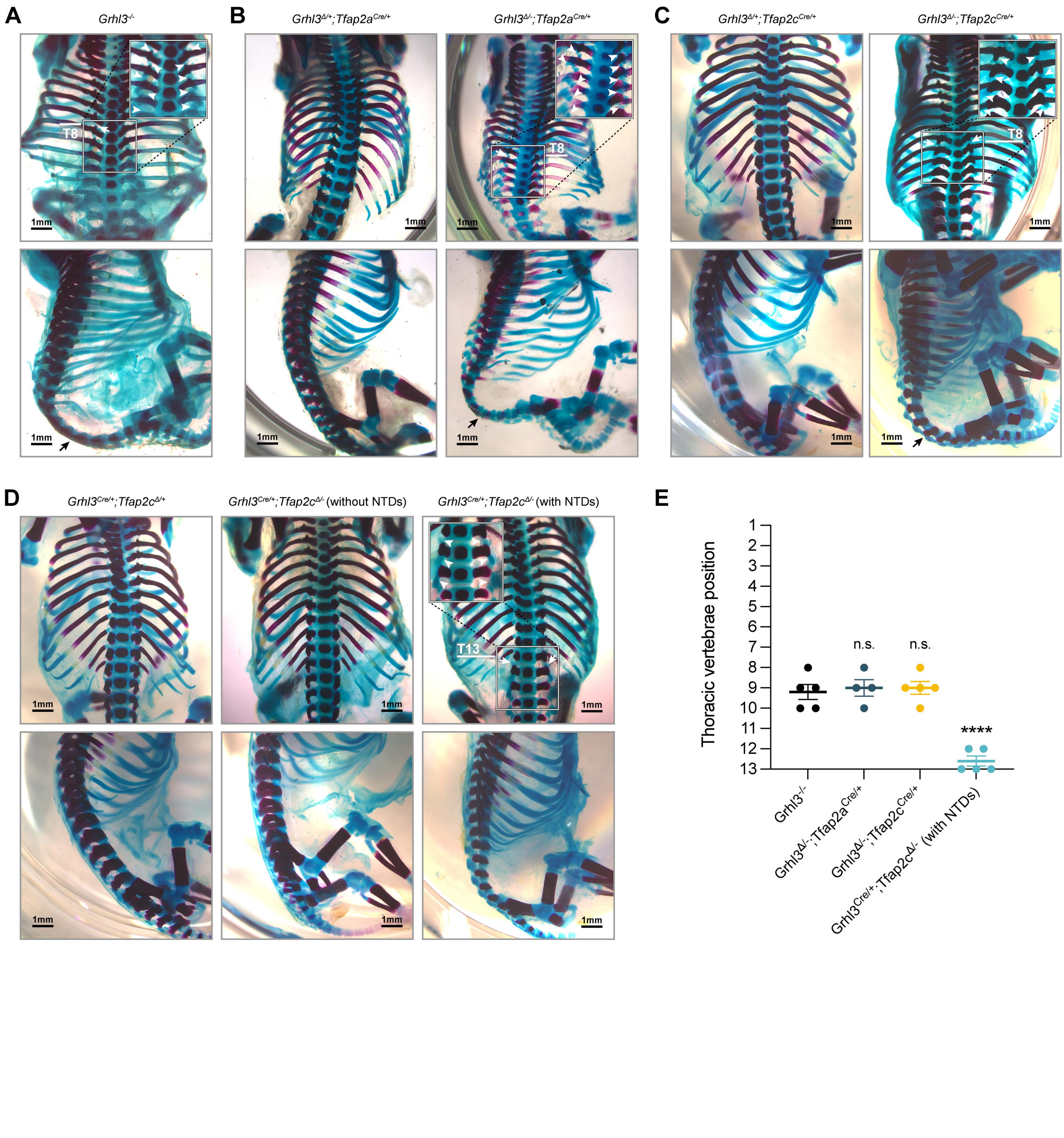

A
Grhl3-/-
T8
1mm
B
Grhl3Δ/+;Tfap2aCre/+
Grhl3Δ/-;Tfap2aCre/+
C
Grhl3Δ/+;Tfap2cCre/+
Grhl3Δ/-;Tfap2cCre/+
D
Grhl3Cre/+;Tfap2cΔ/+
Grhl3Cre/+;Tfap2cΔ/- (without NTDs)
Grhl3Cre/+;Tfap2cΔ/- (with NTDs)
T13
E
Thoracic vertebrae position
n.s.
n.s.
****
Grhl3-/-
Grhl3Δ/-;Tfap2aCre/+
Grhl3Δ/-;Tfap2cCre/+
Grhl3Cre/+;Tfap2cΔ/- (with NTDs)

Figure S4

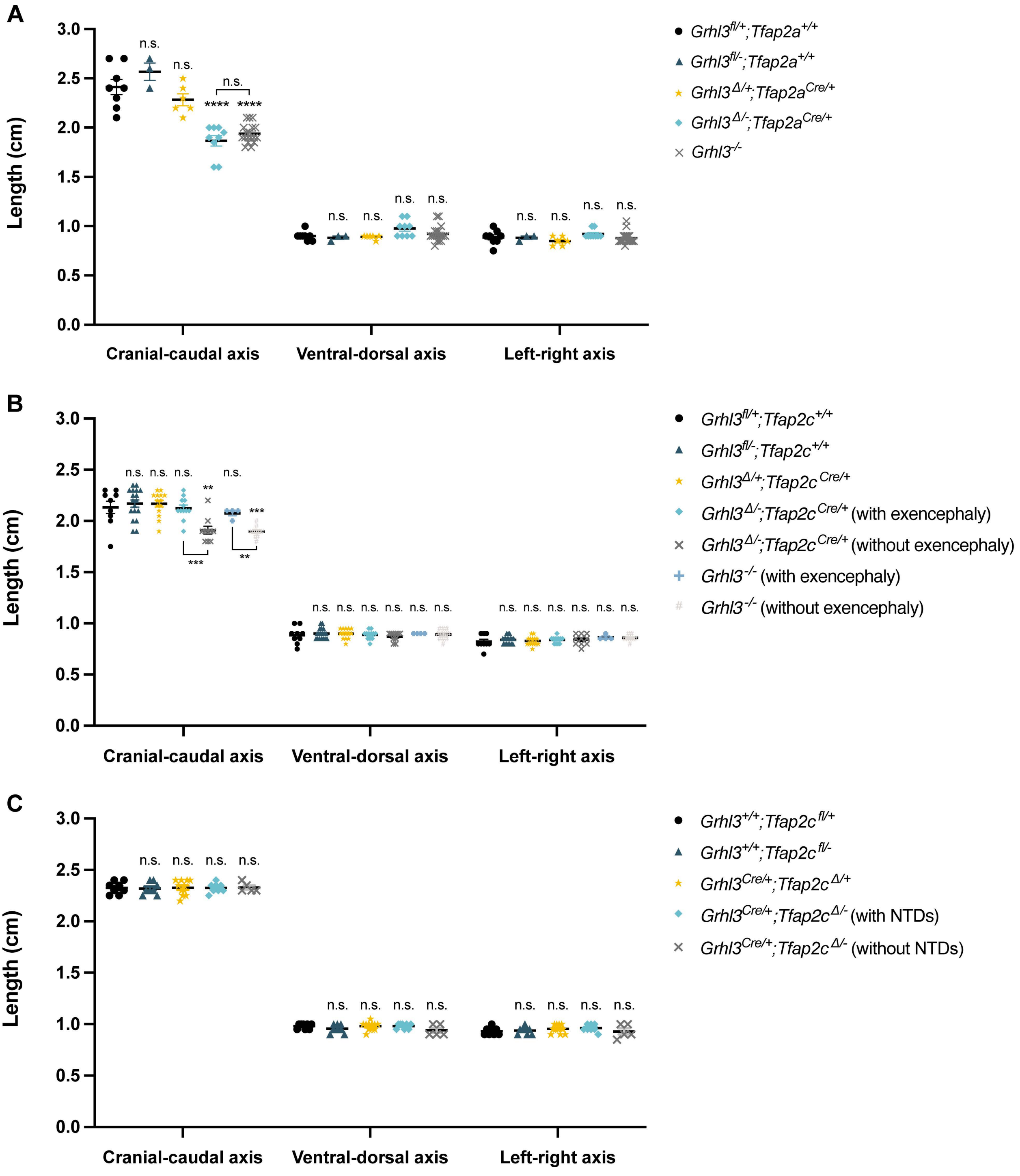
A
Length (cm)
Cranial-caudal axis
Ventral-dorsal axis
Left-right axis
Grhl3fl/+;Tfap2a+/+
Grhl3fl/-;Tfap2a+/+
Grhl3Δ/+;Tfap2aCre/+
Grhl3Δ/-;Tfap2aCre/+
Grhl3-/-
B
Length (cm)
Cranial-caudal axis
Ventral-dorsal axis
Left-right axis
Grhl3fl/+;Tfap2c+/+
Grhl3fl/-;Tfap2c+/+
Grhl3Δ/+;Tfap2cCre/+
Grhl3Δ/-;Tfap2cCre/+ (with exencephaly)
Grhl3Δ/-;Tfap2cCre/+ (without exencephaly)
Grhl3-/- (with exencephaly)
Grhl3-/- (without exencephaly)
C
Length (cm)
Cranial-caudal axis
Ventral-dorsal axis
Left-right axis
Grhl3+/+;Tfap2cfl/+
Grhl3+/+;Tfap2cfl/-
Grhl3Cre/+;Tfap2cΔ/+
Grhl3Cre/+;Tfap2cΔ/- (with NTDs)
Grhl3Cre/+;Tfap2cΔ/- (without NTDs)

Figure S5

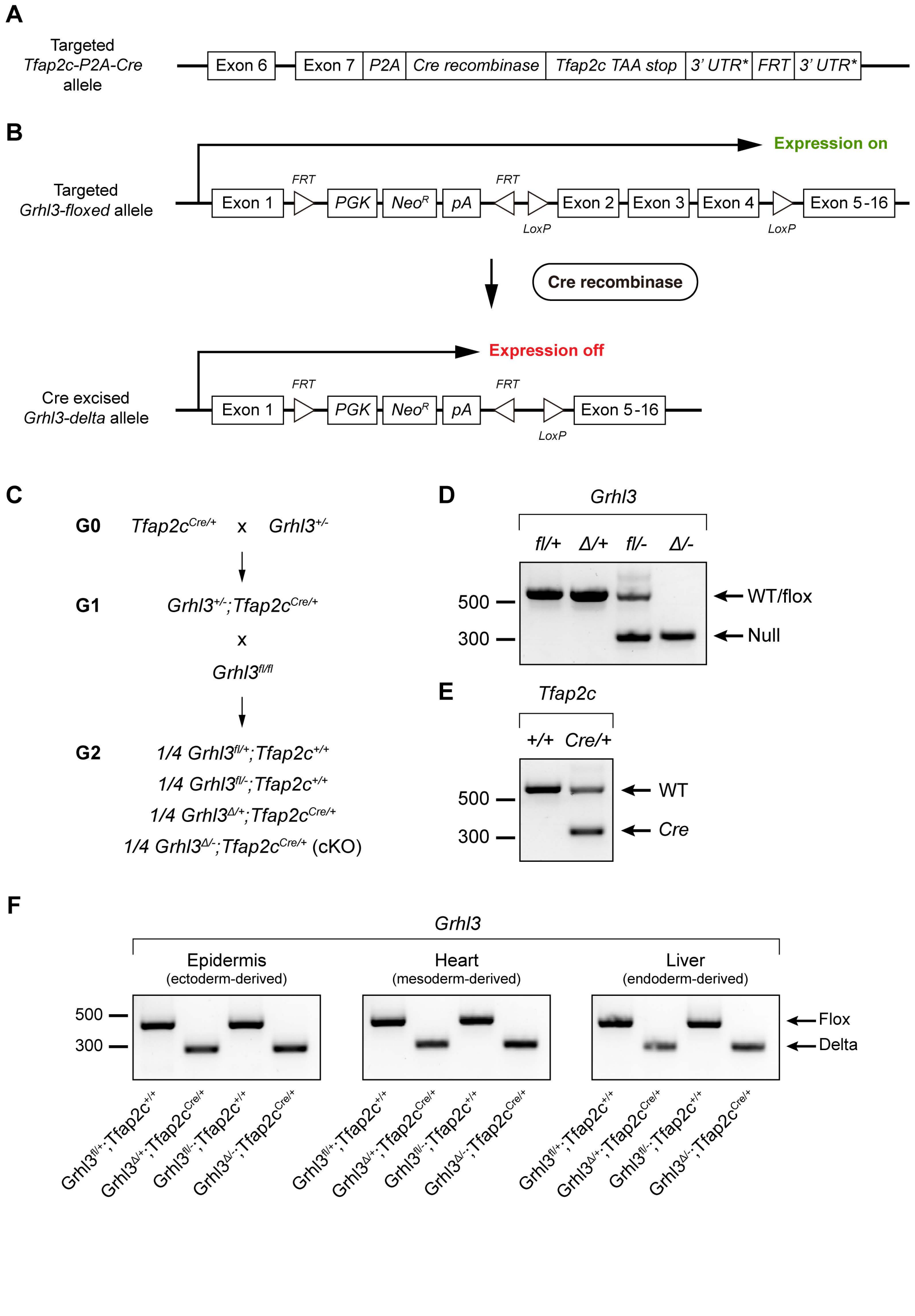

A
Targeted Tfap2c-P2A-Cre allele
Exon 6
Exon 7
P2A
Cre recombinase
Tfap2c TAA stop
3' UTR*
FRT
3' UTR*
B
Expression on
Targeted Grhl3-floxed allele
Exon 1
FRT
PGK
Neo^R
pA
FRT
LoxP
Exon 2
Exon 3
Exon 4
LoxP
Exon 5-16
Cre recombinase
Expression off
Cre excised Grhl3-delta allele
Exon 1
FRT
PGK
Neo^R
pA
FRT
LoxP
Exon 5-16
C
G0 Tfap2c^Cre/+ x Grhl3^+/-
G1 Grhl3^+/-;Tfap2c^Cre/+
x
Grhl3^fl/fl
G2 1/4 Grhl3^fl/+;Tfap2c^+/+
1/4 Grhl3^fl/-;Tfap2c^+/+
1/4 Grhl3^Δ/+;Tfap2c^Cre/+
1/4 Grhl3^Δ/-;Tfap2c^Cre/+ (cKO)
D
Grhl3
fl/+
Δ/+
fl/-
Δ/-
500
300
WT/flox
Null
E
Tfap2c
+/+
Cre/+
500
300
WT
Cre
F
Grhl3
Epidermis
(ectoderm-derived)
Heart
(mesoderm-derived)
Liver
(endoderm-derived)
500
300
Flox
Delta
Grhl3^fl/+;Tfap2c^+/+
Grhl3^Δ/+;Tfap2c^Cre/+
Grhl3^fl/-;Tfap2c^+/+
Grhl3^Δ/-;Tfap2c^Cre/+

Figure S6

A

Genotype	Expected No. of embryos	Observed No. of embryos	P-value	Neural tube phenotype % (n)		
				Curly tail	Spina bifida	Exencephaly
a. E14.5 embryos						
$Grhl3^{fl/+};Tfap2c^{+/+}$	15.5	15	0.90	0 (0)	0 (0)	0 (0)
$Grhl3^{fl/-};Tfap2c^{+/+}$	15.5	16	0.90	0 (0)	0 (0)	0 (0)
$Grhl3^{\Delta/+};Tfap2c^{Cre/+}$	15.5	14	0.70	0 (0)	0 (0)	0 (0)
$Grhl3^{\Delta/-};Tfap2c^{Cre/+}$	15.5	17	0.70	100% (17)	100% (17)	52.9% (9)
Total number from 8 litters	-	62	-	-	-	-
b. E18.5 embryos						
$Grhl3^{fl/+};Tfap2c^{+/+}$	29	28	0.85	0 (0)	0 (0)	0 (0)
$Grhl3^{fl/-};Tfap2c^{+/+}$	29	32	0.58	0 (0)	0 (0)	0 (0)
$Grhl3^{\Delta/+};Tfap2c^{Cre/+}$	29	30	0.85	0 (0)	0 (0)	0 (0)
$Grhl3^{\Delta/-};Tfap2c^{Cre/+}$	29	26	0.58	100% (26)	100% (26)	57.7% (15)
Total number from 17 litters	-	116	-	-	-	-

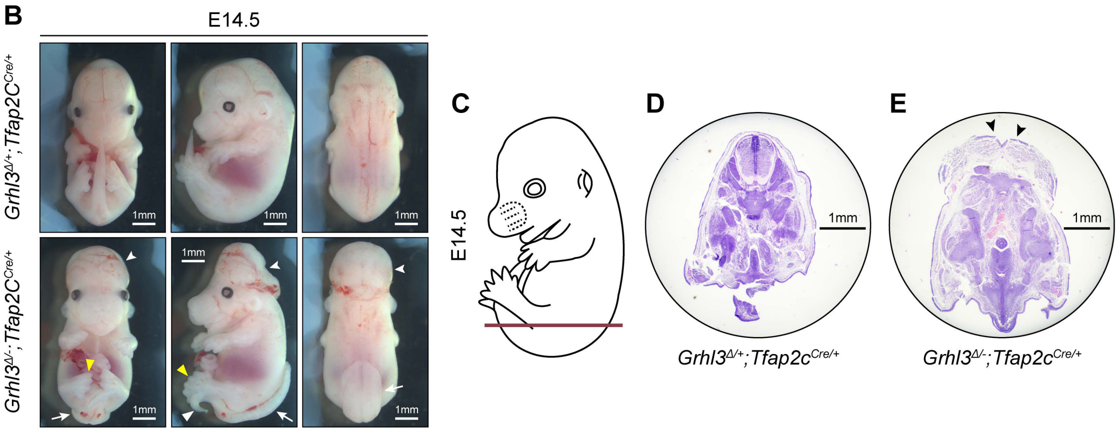

Figure S7

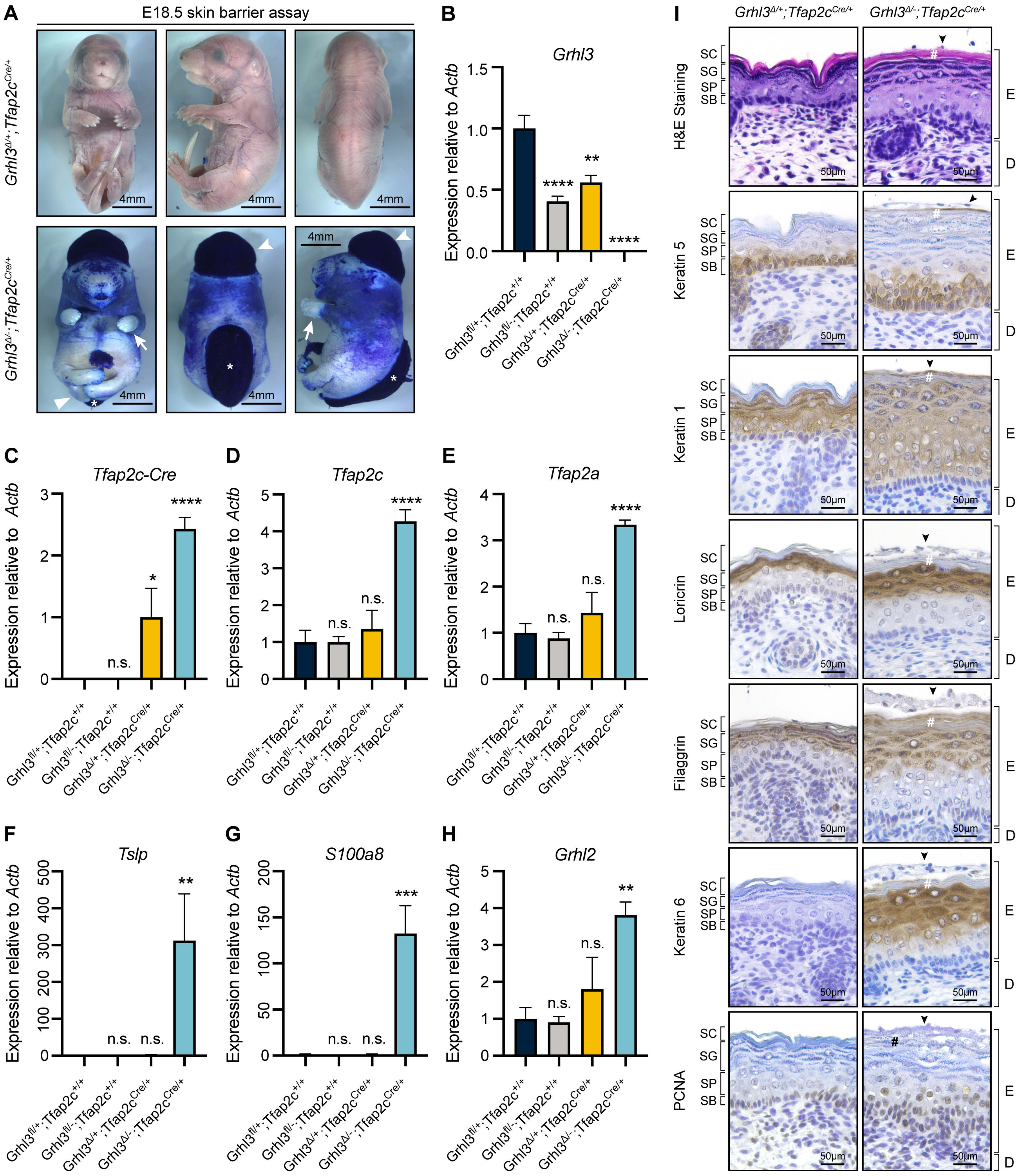
A
E18.5 skin barrier assay
Grhl3Δ/+;Tfap2cCre/+
Grhl3Δ/-;Tfap2cCre/+
4mm
B
Grhl3
Expression relative to Actb
1.5
1.0
0.5
0.0
****
**
****
Grhl3fl/+;Tfap2c+/+
Grhl3fl/-;Tfap2c+/+
Grhl3Δ/+;Tfap2cCre/+
Grhl3Δ/-;Tfap2cCre/+
C
Tfap2c-Cre
n.s.
*
****
D
Tfap2c
n.s.
n.s.
****
E
Tfap2a
n.s.
n.s.
****
F
Tslp
n.s.
n.s.
**
G
S100a8
n.s.
n.s.
***
H
Grhl2
n.s.
n.s.
**
I
Grhl3Δ/+;Tfap2cCre/+
Grhl3Δ/-;Tfap2cCre/+
H&E Staining
Keratin 5
Keratin 1
Loricrin
Filaggrin
Keratin 6
PCNA
SC
SG
SP
SB
E
D
#
50µm

Figure S8

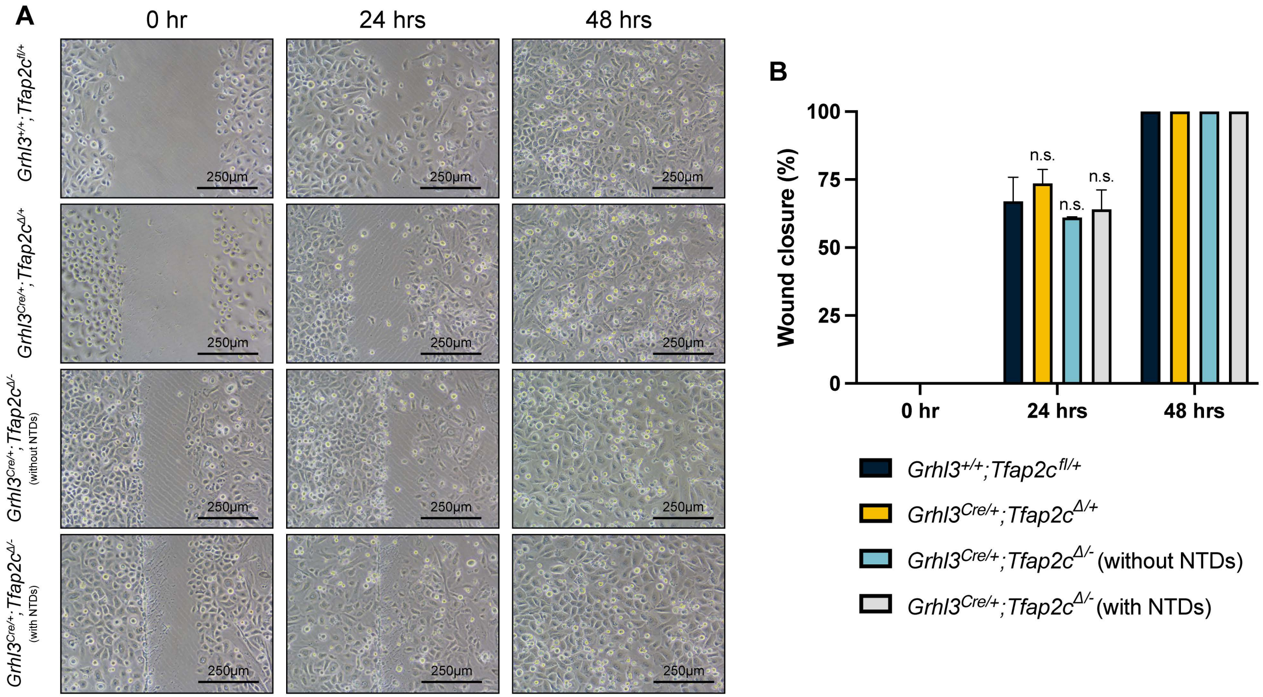
A
0 hr
24 hrs
48 hrs
Grhl3+/+;Tfap2cfl/+
GrhI3Cre/+;Tfap2cΔ/+
Grhl3Cre/+;Tfap2cΔ/- (without NTDs)
Grhl3Cre/+;Tfap2cΔ/- (with NTDs)
250µm
B
Wound closure (%)
100
75
50
25
0
n.s.
0 hr
24 hrs
48 hrs
Grhl3+/+;Tfap2cfl/+
Grhl3Cre/+;Tfap2cΔ/+
Grhl3Cre/+;Tfap2cΔ/- (without NTDs)
Grhl3Cre/+;Tfap2cΔ/- (with NTDs)

Figure S9
